# COVID-19 mRNA vaccine protects against SARS-CoV-2 Omicron BA.1 infection in diet-induced obese mice through boosting host innate antiviral responses

**DOI:** 10.1016/j.ebiom.2023.104485

**Published:** 2023-02-27

**Authors:** Yanxia Chen, Wenchen Song, Can Li, Jiaxuan Wang, Feifei Liu, Zhanhong Ye, Peidi Ren, Yihan Tong, Junhua Li, Zhihua Ou, Andrew Chak-Yiu Lee, Jian-Piao Cai, Bosco Ho-Yin Wong, Jasper Fuk-Woo Chan, Kwok-Yung Yuen, Anna Jin-Xia Zhang, Hin Chu

**Affiliations:** a Department of Microbiology, State Key Laboratory of Emerging Infectious Diseases, Carol Yu Centre for Infection, School of Clinical Medicine, Li Ka Shing Faculty of Medicine, The University of Hong Kong, Pokfulam, Hong Kong Special Administrative Region, People's Republic of China; b Centre for Virology, Vaccinology and Therapeutics, Hong Kong Science and Technology Park, Hong Kong Special Administrative Region, People's Republic of China; c Shenzhen Key Laboratory of Unknown Pathogen Identification, BGI-Shenzhen, Shenzhen, 518083, People's Republic of China; d Department of Infectious Disease and Microbiology, The University of Hong Kong-Shenzhen Hospital, Shenzhen, Guangdong Province, People's Republic of China; e Department of Microbiology, Queen Mary Hospital, Pokfulam, Hong Kong Special Administrative Region, People's Republic of China; f Academician Workstation of Hainan Province, Hainan Medical University-The University of Hong Kong Joint Laboratory of Tropical Infectious Diseases, Hainan Medical University, Haikou, Hainan Province, People’s Republic of China; and The University of Hong Kong, Pokfulam, Hong Kong Special Administrative Region, China

**Keywords:** Obesity, Diet-induced obese mouse, SARS-CoV-2, COVID-19, Vaccination, Omicron

## Abstract

**Background:**

Obesity is a worldwide epidemic and is considered a risk factor of severe manifestation of Coronavirus Disease 2019 (COVID-19). The pathogenicity of severe acute respiratory syndrome coronavirus 2 (SARS-CoV-2) and host responses to infection, re-infection, and vaccination in individuals with obesity remain incompletely understood.

**Methods:**

Using the diet-induced obese (DIO) mouse model, we studied SARS-CoV-2 Alpha- and Omicron BA.1-induced disease manifestations and host immune responses to infection, re-infection, and COVID-19 mRNA vaccination.

**Findings:**

Unlike in lean mice, Omicron BA.1 and Alpha replicated to comparable levels in the lungs of DIO mice and resulted in similar degree of tissue damages. Importantly, both T cell and B cell mediated adaptive immune responses to SARS-CoV-2 infection or COVID-19 mRNA vaccination are impaired in DIO mice, leading to higher propensity of re-infection and lower vaccine efficacy. However, despite the absence of neutralizing antibody, vaccinated DIO mice are protected from lung damage upon Omicron challenge, accompanied with significantly more IFN-α and IFN-β production in the lung tissue. Lung RNAseq and subsequent experiments indicated that COVID-19 mRNA vaccination in DIO mice boosted antiviral innate immune response, including the expression of IFN-α, when compared to the nonvaccinated controls.

**Interpretation:**

Our findings suggested that COVID-19 mRNA vaccination enhances host innate antiviral responses in obesity which protect the DIO mice to a certain degree when adaptive immunity is suboptimal.

**Funding:**

A full list of funding bodies that contributed to this study can be found in the Acknowledgements section.

**Research in context**

**Evidence before this study**

We searched PubMed in May 2022, with no starting date limitations, using the terms “SARS-CoV-2” and “Omicron BA.1 or Alpha” and “Diet-induced obese mouse” and “COVID-19 mRNA vaccination” for articles in English. Our search did not reveal any report investigated in SARS-CoV-2 Omicron BA.1 and Alpha infection and COVID-19 mRNA vaccination of wild type diet-induced obese mouse for both in vitro and in vivo studies.

**Added value of this study**

In this study, we demonstrate that the pathogenicity of SARS-CoV-2 Omicron BA.1 is similar in DIO mice when compared with Alpha, despite results in milder diseases in lean mice. Next, we reveal that DIO mice are more susceptible to SARS-CoV-2 re-infection and are less efficiently protected by COVID-19 mRNA vaccine due to impaired adaptive immune response. However, after two doses of COVID-19 mRNA vaccination, the lower respiratory tract of vaccinated DIO mice is largely protected from Omicron BA.1 infection despite undetectable serum neutralizing antibody in DIO mice. Both in vivo and in vitro studies suggest the mRNA vaccine may protect DIO mice from SARS-CoV-2 infection by improving the host innate immune responses including the type I interferon signaling responses.

**Implications of all the available evidence**

These findings suggest that obesity increases the susceptibility of SARS-CoV-2 re-infection and vaccine breakthrough infections due to impaired adaptive immune responses. Nevertheless, COVID-19 mRNA vaccination offers partial protection in DIO mice by boosting the host innate immune responses.

## Introduction

Coronavirus Disease 2019 (COVID-19) is caused by severe acute respiratory syndrome coronavirus-2 (SARS-CoV-2), which has resulted in more than 630 million infections with over 6.5 million deaths.^1^, ^2^, ^3^ Advanced age and comorbidities including hypertension, obesity, certain cancers, and cardiovascular diseases have been suggested as risk factors of developing severe COVID-19.^4^ Among these conditions, obesity is currently considered a worldwide epidemic and is associated with type II diabetes mellitus, cardiovascular disease, hypertension, and nonalcoholic fatty liver disease due to the state of chronic low-grade systemic inflammation.^5^ Previous studies reported that diet-induced obese (DIO) mice mounted dysregulated innate immune responses and developed severe lung damage after pandemic influenza H1N1 infection.^6^, ^7^, ^8^ Since the outbreak of COVID-19 pandemic, clinical reports suggested that people with obesity-related conditions are at higher risk of COVID-19 and are more prone to severe illness.^9^^,^^10^ Additionally, a recent study indicated that obesity is associated with delayed SARS-CoV-2 clearance and unfavorable prognosis in COVID-19 patients.^11^ However, the pathogenicity of SARS-CoV-2 and host responses to re-infection and vaccination in people with obesity remain incompletely understood.^12^

Since its emergence in 2019, SARS-CoV-2 continues to evolve and generate new SARS-CoV-2 variants of concerns (VOCs) including Alpha, Beta, Gamma, Delta, and Omicron. These VOCs carry key mutations in spike and other viral proteins that collectively modulate their pathogenesis, immune evasiveness, and transmissibility. In late November of 2021, SARS-CoV-2 Omicron BA.1 was first reported in South Africa, which rapidly spread and replaced Delta as the dominantly circulating SARS-CoV-2 variant in early 2022. Omicron BA.1 contains over 30 amino acid changes in spike in comparison to the ancestral SARS-CoV-2, which results in its unique virological features including reduced pathogenicity, elevated transmissibility^13^, ^14^, ^15^, ^16^, ^17^ and evasion of vaccine induced immunity.^18^, ^19^, ^20^ Since Omicron and its sublineages are now the predominant SARS-CoV-2 strains, it is important to understand the manifestation of Omicron infection in the obese population. Most importantly, the effectiveness of COVID-19 mRNA vaccination in obese state remains incompletely explored.

In this study, we used DIO mice as an animal model to simulate the manifestation of Alpha and Omicron BA.1 in the context of SARS-CoV-2 pathogenesis, re-infection, and COVID-19 mRNA vaccine-mediated protection in the obese individuals. First, we found that SARS-CoV-2 resulted in more severe disease outcomes in DIO mice than in lean mice. Meanwhile, we found that serum neutralizing antibody responses and viral specific T cell interferon-γ responses induced by either virus infection or COVID-19 mRNA vaccination were severely attenuated in DIO mice, which resulted in higher susceptibility to re-infection and less COVID-19 mRNA vaccine protection of DIO mice when compared with lean mice. Second, although Omicron BA.1 infection caused attenuated diseases in lean mice, it resulted in severe disease in DIO mice similar to that of Alpha. Third, we showed that two-dose of COVID-19 mRNA vaccinations failed to induce serum neutralizing antibody against Omicron BA.1 but was capable of ameliorating Omicron BA.1-induced lung damage in DIO mice. Transcriptomics studies of lung tissues demonstrated that the innate immune antiviral responses in DIO mice were significantly upregulated by vaccination, indicating that the COVID-19 mRNA vaccination offered protection to DIO mice through boosting host innate antiviral immunity. Altogether, our study revealed important knowledge of SARS-CoV-2 Alpha and Omicron BA.1 infection, re-infection, and COVID-19 mRNA vaccination in DIO mice.

## Methods

### Ethics statement

All experiments involving live SARS-CoV-2 were performed in the Biosafety Level-3 facility at Department of Microbiology, the University of Hong Kong (HKU) according to the standard operating procedures. All the animal experimental procedures were approved by the Committee on the Use of Live Animals in Teaching and Research of the HKU under CULATR 5786-21 and compliance with animal use guidelines.

### Virus and biosafety

SARS-CoV-2 B.1.1.7/Alpha (GISAID: EPI_ISL_1273444) and B.1.1.529.1/Omicron BA.1 (GenBank:OM212469)^18^ were isolated from laboratory-confirmed COVID-19 patients in Hong Kong. Alpha and Omicron BA.1 were cultured and titrated by plaque assay in VeroE6-TMPRSS2 cells and stored at −80 °C before use.

### Animals

C57BL/6N mice were obtained from the Center of Comparative Medicine Research of HKU and kept in BSL-2 animal laboratory with a 12-h-interval day/night cycle.^21^ Obesity induction was performed as described previously.^6^ 3 weeks-old newly weaned female mice were randomly divided into 2 groups, one group was fed with 45 Kcal% high-fat diet (D12451, Research Diet Inc, New Brunswick, N. J.) for 20 weeks to induce diet-induced obese (DIO) mice and the control group was fed with standard pellet food containing 13.2 Kcal% diet (PicoLab Rodent Diet 20, LabDiet Code 5053, PMI) as lean mice. Average body weight of DIO mouse used in this study was 40–50 g and the control lean mice group was 25–30 g.

### Cell culture and stimulation

Alveolar macrophages (AMs) were isolated by bronchoalveolar lavage (BAL). Briefly, After the mice were sacrificed by intra-peritoneal injection of pentobarbital, the lungs were flushed with 1 mL cool PBS for three times to obtain 3 mL bronchoalveolar lavage fluid (BALF). BALF from mice in the same group were pooled and centrifuged at 500×*g* for 10 min at 4 °C to collect precipitated cells, AMs were seeded with a density of 50,000 cells/well in 96-well plates cultured in RPMI 1640 plus 1% penicillin-streptomycin at 37 °C in a moist atmosphere of 5% CO_2_ for 2 h, then monolayers of AMs could adhere to culture plates, nonadherent cells were washed from plates with PBS and AMs were cultured in RPMI 1640 plus 1% penicillin-streptomycin and 10% fetal bovine serum (FBS) with or without stimulation of 1 μg/mL mRNA vaccine, 100 μg/mL Poly (I:C) or 100 ng/mL spike protein for 24 h.^22^ Supernatants were collected for ELISA test. Cells were washed thoroughly with PBS and harvested for RNA extraction.

### Alpha and Omicron BA.1 challenge in mice

DIO and lean mice under anesthesia by Ketamine (100 mg/kg) and Xylazine (10 mg/kg)^23^ were intranasal inoculation with 10^3^ PFUs of Alpha or Omicron BA.1 diluted in 20 μl phosphate buffered saline (PBS), control mice were inoculated with the same volume of PBS. Body weight and symptoms of the infected mice were monitored for 14 days, the symptoms of disease including ruffled fur, hunched posture and labored breathing and one score was given to each sign. Mice were sacrificed by intra-peritoneal injection of pentobarbital at 2 and 4 days post infection (dpi), 3–6 mice in each group were euthanized to harvest blood samples, lung and nasal turbinate tissues for virological, histopathological and immunological assessment. Lung samples from each mouse were collected for the assessments. Each data dot in the figure panel represents result from one mouse.

### Vaccination procedure

Mice were randomly divided into groups and given a two-dose regimen of COVID-19 mRNA vaccination (BNT162b2, lot number 1B004A, BioNTech, Germany) at 14-day interval. The vaccine is based on a nucleoside-modified mRNA formulated in lipid nanoparticles that encodes the ancestral SARS-CoV-2 full-length. DIO and lean mice were intramuscularly injected with 50 μl (5 μg) of COVID-19 mRNA vaccine or normal saline as the control.^24^^,^^25^ At day 14 after the first dose of vaccination, we collected blood samples and then provided the second dose of mRNA vaccine. At 14 days after the second dose, blood samples were collected for assessment of antibody responses. Mice were subsequently intranasally inoculated with 10^3^ PFU of Alpha or Omicron BA.1. At 2 dpi, mice were sacrificed by intra-peritoneal injection of pentobarbital and samples including blood samples, lung and nasal turbinate tissues were collected for virological, histological and immunological assessment. Lung tissues were separated into three parts, the left lung was harvested for histological assessment, the caudal lobe of the right lung was collected as lung homogenate and the rest of right lung for RNA extraction.

### Determination of SARS-CoV-2 gene copies and infectious viral titre in lung and nasal turbinate tissues

Total RNA from lung and nasal turbinate (NT) tissues of mice with Alpha and Omicron BA.1 infection were extracted by the MiniBEST Universal RNA extraction kit (9766, Takara Bio Inc. Shiga, Japan). SARS-CoV-2 RNA-dependent RNA polymerase (RdRp) gene copies were quantified by a QuantiNova Probe RT-PCR Kit (208354, Qiagen). qRT-PCR for detection of SARS-CoV-2 RdRp gene copies and the house-keeping gene β-actin for normalization were performed on a LightCycler 96 system (Roche Applied Sciences, Indianapolis, USA).^21^ For infectious virus titration, homogenates from lung (caudal lobe of the right lung) and NT tissues infected with Alpha and Omicron BA.1 were performed by a 50% tissue culture infectious dose (TCID_50_) assay in VeroE6-TMPRSS2 cells.^26^ Homogenates were 10-fold serial dilution and incubated with VeroE6-TMPRSS2 cells in 96-well plate for 1 h at a 37 °C incubator, supernatants were discarded and cells were further incubated in 37 °C for 72 h. Cytopathic effect (CPE) was observed and 50% tissue infectious titre were determined by the Reed & Munch endpoint calculation method.^26^

### Histopathology, immunohistochemistry, and immunofluorescence staining of lung and nasal turbinate tissue sections

Haematoxylin and Eosin (H&E) staining of formalin-fixed and paraffin-embedding tissue sections (4 μm each) were observed for histopathology changes. Severity of histopathology in lungs were given score under complete masking^27^ by assessment of pulmonary congestion, interstitial infiltration, alveolar infiltration, hemorrhage and scored 0–4 as described previously.^26^ The following criteria were used for scoring: 0, normal lung section; 1, blood vessel congestion, perivascular or peribronchiolar infiltration; 2, in addition to 1 with diffuse alveolar wall congestion and infiltration; 3, air space infiltration, exudation, hemorrhage of localized alveolitis; 4, diffuse alveolitis were observed. Immunohistochemistry staining was performed by a DAB (3,3′-diaminobenzidine) substrate kit (Vector Laboratories) as we previously described.^28^ Briefly, For ACE2 antigen detection, ACE2 recombinant rabbit monoclonal antibody (MA5-32307, Invitrogen) were used and followed with color development by using the DAB substrate kit. The ACE2 protein was detected by haematoxylin and then mounted the tissue sections with the VectaMount permanent mounting medium (Vector Laboratories). For SARS-CoV-2 antigen expression, slides of lung and NT tissues were stained with an in-house antibody of rabbit anti SARS-CoV-2 nucleocapsid protein (NP) followed by a secondary antibody of FITC–conjugated goat anti rabbit IgG (65-6111, Thermo Fisher Scientific, Waltham, MA, USA). The following criteria were used for NP scoring. Lung: “score 0”- no fluorescence staining signal; “score 1”- only in 1–3 bronchiolar epithelium with N antigen positive cells; “score 2”- more than 3 bronchiolar epithelium with N antigen positive cells; “score 3”- Bronchiolar epithelium with a few positive cells in nearby alveolar; “score 4”- multiple foci or large area of alveoli with N antigen positive cells. NT: “score 0”- no fluorescence staining signal; “score 1”- a few N antigen positive cells scattered in the epithelium; “score 2”- epithelium showing continually positive N antigen focus in adjacent cells; “score 3”- more N antigen positive of epithelial foci distributed in different area. Images were captured by using microscope of Olympus BX53 semi-motorized fluorescence or bright-field with OLYMPUS CellSense Standard Software.

### RNA isolation and real-time reverse-transcription polymerase chain reaction

Total RNA was extracted from tissue homogenates (the cranial, middle and accessory lobes were harvested for lungs) and reverse-transcription for cDNA was performed by MiniBEST Universal RNA Extraction Kit and RT Reagent Kit (RR036A Takara Bio Inc.) following the manufacturer's instruction. Expression level of cytokines, chemokines, and interferons were detected by qRT-PCR with specific primers (Supplementary Table S1) using a SYBR Premix Ex Taq II Kit (RR820A, Takara Bio Inc.). Values of each gene were normalized with house-keeping gene β-actin and presented as 2^−ΔCt^ as we previously described.^28^^,^^29^

### Enzyme-linked immunosorbent assay (ELISA)

SARS-CoV-2 nucleoprotein (N), spike protein receptor-binding domain (RBD) and inactivated SARS-CoV-2 were coated in 96-well immunoplates (Nunc-Immuno Modules; Nunc A/S, Roskilde, Denmark) in 0.05 M NaHCO_3_ and incubated at 4 °C, overnight. Serum samples were 2-fold serially diluted and added to the coated plate, incubated at 4 °C for 1 h followed by horseradish peroxidase (HRP)-conjugated secondary antibodies (Rabbit anti-mouse IgG, Goat anti-mouse IgG1, IgG2a, IgG2b, ab6728, ab98693, M32307, M32507, Abcam and Invitrogen) at 37 °C for 1 h. Color development was performed with 3,3′,5,5′-tetramethylbenzidine solution (#N301, Thermo Fisher Scientific) at 37 °C for 15 min and stop with H_2_SO_4_. The optical density (OD) values were read at 450 nm. Antibody titres were determined by a cut off OD value which was set at the mean OD of uninfected serum at all dilutions plus 3 standard deviations, and the highest dilution which produces an OD value above the cut off was determined as the antibody titre of serum.^30^ IFN-α, IFN-β, Albumin and hemoglobin concentrations were determined using a mouse IFN-α (Invitrogen, USA), IFN-β (R&D systems, USA), albumin and hemoglobin (Abcam, Cambridge, UK) ELISA kit following the manufacturer's instructions.

### Microneutralization (MNT) assay

Serum samples were serially diluted 2-fold starting from 1:10 in PBS and mixed with 100 TCID _50_ of SARS-CoV-2 for 1 h at 37 °C, the mixture was added into pre-seeded VeroE6-TMPRSS2 cells in 96-well plate at 37 °C for 72 h. CPE was observed and neutralizing antibody titres were determined as the highest dilution of serum that completely inhibited the cytopathic effect.

### Enzyme-linked immunospot (ELISpot) assay

Virus-specific IgG producing cells were detected by seeding single cell (2.5 × 10^5^ cells/per well) suspension of lung and spleen tissues into ELISpot plate with inactivated SARS-CoV-2 (5 μg/mL) at 37 °C for 48 h. IgG-producing cells were determined with alkaline phosphatase (AP) conjugated-goat anti mouse IgG antibody (62-6522, Invitrogen).^31^ For Virus-specific IFN-γ producing cells detection, 2.5 × 10^5^ cells/per well single cell suspension of lung and spleen tissues were incubated in IFN-γ ELISpot plate stimulating with SARS-CoV-2 RBD peptide pool and N protein at 37 °C for 48 h, IFN-γ producing cells were determined using a mouse IFN-γ ELISpot BASIC kit (3321-2A, Mabtech, Inc., Stockholm, Sweden) following the manufacturer's instructions.^32^

### RNA sequencing and data analysis

Total RNA from lung tissue cells of DIO and lean mice (n ≥ 3 per group) was isolated using NucleoSpin RNA Kit (740955.250, MACHEREY-NAGEL, Duren, Germany). DNA-depleted and purified RNA was used to construct double-stranded (ds) cDNA library using MGIEasy RNA Library preparation reagent set (MGI, Shenzhen, China) following the standard protocol. Sequencing data were filtered with fastp v0.20.1 to remove adapter and low quality reads.^33^ Ribosomal RNA (rRNA) reads was filtered with URMAP v1.0.1480 (Edgar, 2020),^34^ HISAT2 v2.2.0 was used to map the reads against the mouse reference genome (GRCm38/ENSEMBL 84).^35^ The alignment file was used for assembling transcripts, estimating their abundances, and detecting differential expression of genes, the gene expression levels were quantified by StringTie v2.1.5.^36^ Principal components analysis (PCA) was conducted with R v4.0. Differentially expressed genes (DEGs) and determined based on gene counts with DESeq2 v3.15.^37^ DEGs between different treatment groups were identified by clusterProfile with the threshold of |log_2_FC| > 1 and FDR value < 0.05, and used for enrichment analysis involving Gene Ontology (GO), the Kyoto Encyclopedia of Genes and Genomes Pathway (KEGG).^38^ All genes in the mouse genome were used as the enrichment background. ImmuCellAI_Mouse was used to determine the immune cell abundance based on the RNAseq data.^39^

### Statistical analysis

Data represented means and standard deviations. Statistical differences between two groups were evaluated with Student's t-test using GraphPad Prism 8. Statistical differences between three or more groups were evaluated with one-way or two-way ANOVA using GraphPad Prism 9. Differences were considered statistically significant when p < 0.05. The figures and graphs in the manuscript were prepared with GraphPad Prism 8, Adobe Illustrator, or BioRender.com.

### Reagent validation

Antibodies used in the study have been validated by the commercial source that they are purchased from. Detailed information of reagents used in the study can be found in the Reagent Validation file of Supplemental Data (Supplementary Table S2).

### Role of funders

The funding sources had no role in study design, data collection, analysis or interpretation or writing of the report.

## Results

### SARS-CoV-2 Alpha and Omicron BA.1 result in more severe diseases in DIO mice than in lean mice

**Fig. 1:**
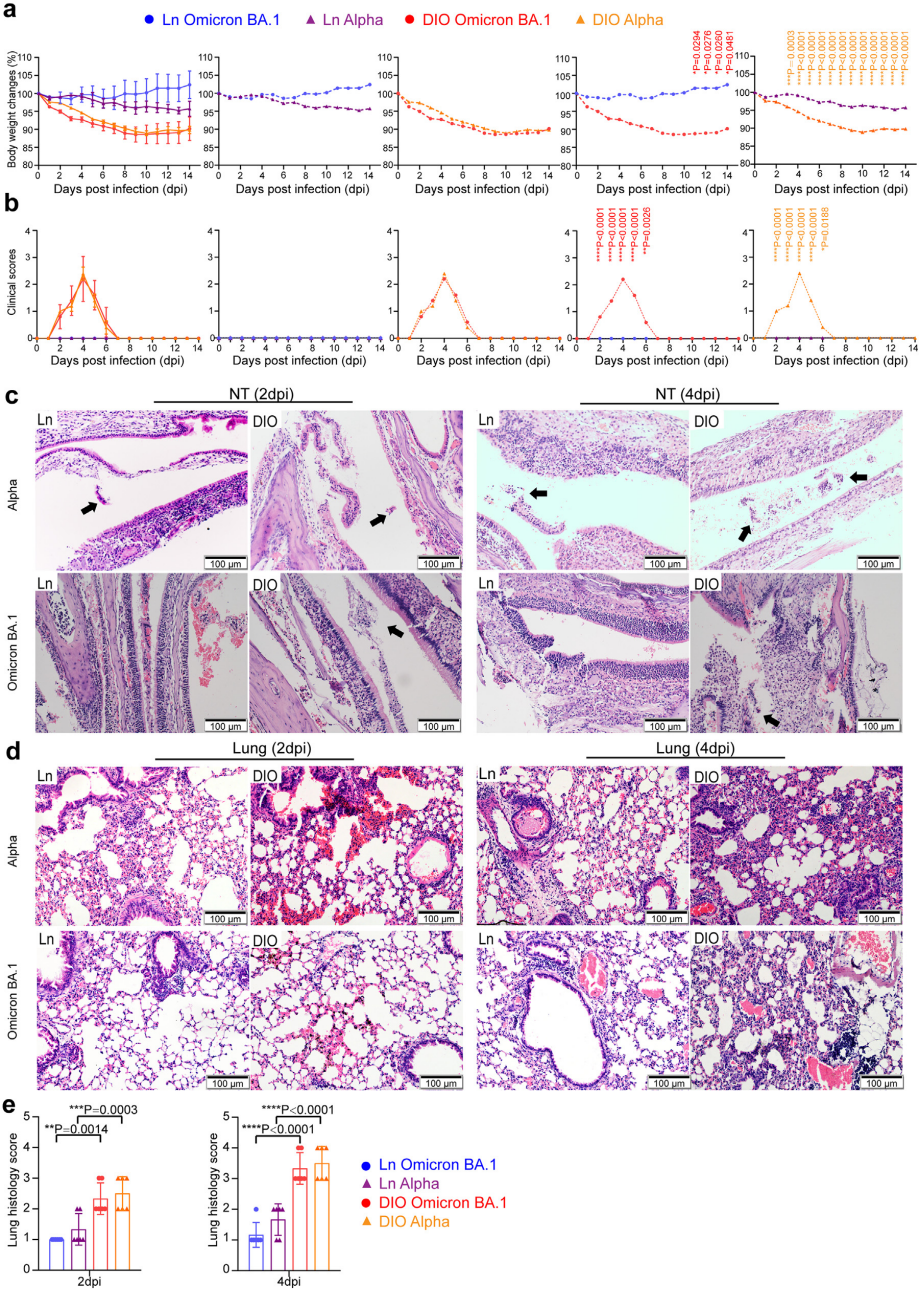
**SARS-CoV-2 Alpha and Omicron BA.1 cause more severe diseases in DIO mice than in lean mice.** Diet-induced obese (DIO) and lean (Ln) mice were intranasally inoculated with 10^3^ PFU of Alpha and Omicron BA.1. Body weight and signs of disease of the infected mice were monitored for 14 days. Lung and nasal turbinate (NT) tissues were collected at day 2 and day 4 post infection (n = 6 in each group). **a** Body weight changes in DIO and Ln mice infected with Alpha or Omicron BA.1. **b** Clinical scores of disease signs upon virus infection. A score of 1 was given to each disease sign (ruffled fur, hunched back and laboured breathing). Highest total score = 3 for each mouse. **c** and **d** Representative images of hematoxylin and eosin (H&E) staining for NT sections (**c**) and lung sections (**d**) of mice infected with Alpha or Omicron BA.1 at 2 and 4 dpi. The arrows indicated nasal epithelium destruction and detachment into the nasal cavity. **e** Quantification of histopathological damage in lung sections at 2 and 4 dpi. Images in (**c** and **d**) are representative images from 6 mice, n = 6 in each group. dpi, days post infection; NT, nasal turbinate. Data represented means and standard deviations from the indicated number of biological repeats. Statistical significance between groups was determined with one way-ANOVA (**e**), two-way ANOVA (**a** and **b**). ∗ represented p < 0.05, ∗∗ represented p < 0.01, ∗∗∗ represented p < 0.001, ∗∗∗∗ represented p < 0.0001. Scale bar in (**c** and **d**) represented 100 μm.

To understand the pathogenicity of SARS-CoV-2 in the context of obesity, DIO and lean mice were inoculated with 10^3^ PFU of Omicron BA.1 (B.1.1.529.1) or Alpha (B.1.1.7) via the intranasal route. Omicron BA.1 and Alpha carry the N501Y substitution in spike that allow them to infect wild-type mice.^40^^,^^41^ In a 14-day disease course, we observed a slow but constant decline of body weight in Alpha-infected lean mice with a mean body weight loss of 5% at 14 dpi (Fig. 1a). In contrast, we observed a mean body weight increase of 1.5% in Omicron BA.1-infected lean mice, indicating that the pathogenicity of Omicron BA.1 in lean mice was significantly attenuated (Fig. 1a), which is in keeping with recent studies from us and others.^14^^,^^42^ However, infection of both Alpha and Omicron BA.1 resulted in more severe body weight loss in diet-induced obese (DIO) mice, which was approximately 12% at 9 dpi when compared with their original body weight (100%) at 0 dpi (Fig. 1a). Interestingly, while there was a clear difference in the mean body weight change between Alpha- and Omicron BA.1-infected lean mice (14 dpi: −5.0% vs +1.5%), the body weight loss of Alpha- and Omicron BA.1-infected DIO mice were largely the same (14 dpi: −11.1% vs −11.4%) (Fig. 1a). Consistent with the body weight measurements, Alpha and Omicron BA.1 infection resulted in comparable clinical symptoms in DIO mice, including ruffled fur, hunched back, and labored breathing that peaked at 4 dpi (Fig. 1b), while we did not observe any sign of disease in lean mice upon Alpha or Omicron BA.1 infection. Next, we assessed the histological changes in upper and lower respiratory tissues. Mock-infected mouse nasal turbinate (NT) and lung tissues were shown as control (Supplementary Fig. S1). We observed mild virus-induced epithelium destruction and inflammatory infiltration in NT of both Alpha- and Omicron BA.1-infected lean mice at 2 dpi. The epithelial desquamation and immune infiltration were more dramatic in the NT sections of DIO mice infected with Alpha or Omicron BA.1 at 2 dpi when compared to lean mice (Fig. 1c). At 4 dpi, mild NT epithelium destruction with a few luminal cell debris was detected in Alpha-infected lean mice, while NT of Omicron BA.1-infected lean mice appeared relatively intact. However, we continued to detect severe epithelial destruction, luminal debris and submucosal immune cells infiltration in the NT sections of DIO mice at day 4 after Alpha or Omicron BA.1 infection (Fig. 1c). In the lung tissues of lean mice, Alpha infection resulted in localized interstitial inflammation and mild alveolar capillary congestion at 2 and 4 dpi, while Omicron BA.1 infection resulted in lung interstitial inflammation at 2 dpi but was largely resolved at 4 dpi (Fig. 1d). In sharp contrast, more severe histological damages of alveoli were observed in the lung tissues of DIO mice after both Alpha or Omicron BA.1 infection, which manifested as severe pulmonary blood vessel congestion at 2 dpi. Increased peribronchiolar and peri-vascular immune cell infiltration, as well as immune cells and fluid exudates were observed in alveolar sacs at 4 dpi (Fig. 1d). The concentration of albumin in the bronchoalveolar lavage fluid was significantly higher in DIO mice than in lean mice at day 4 after Alpha infection, suggesting increased alveolar capillary permeability in SARS-CoV-2-infected DIO mice (Supplementary Fig. S2). Consistent with these findings, semi-quantitative histology assessment of lung tissues indicated that infection of both Omicron BA.1 and Alpha resulted in more severe lung histopathology in DIO mice than that in lean mice (Fig. 1e). Overall, these results indicate that SARS-CoV-2 infection results in more severe disease manifestations in DIO mice than in lean mice. Importantly, while Omicron BA.1 is less pathogenic than Alpha in lean mice, the two SARS-CoV-2 variants are more pathogenic and cause similar diseases in DIO mice.

### SARS-CoV-2 replicates more efficiently in DIO mice than in lean mice

**Fig. 2:**
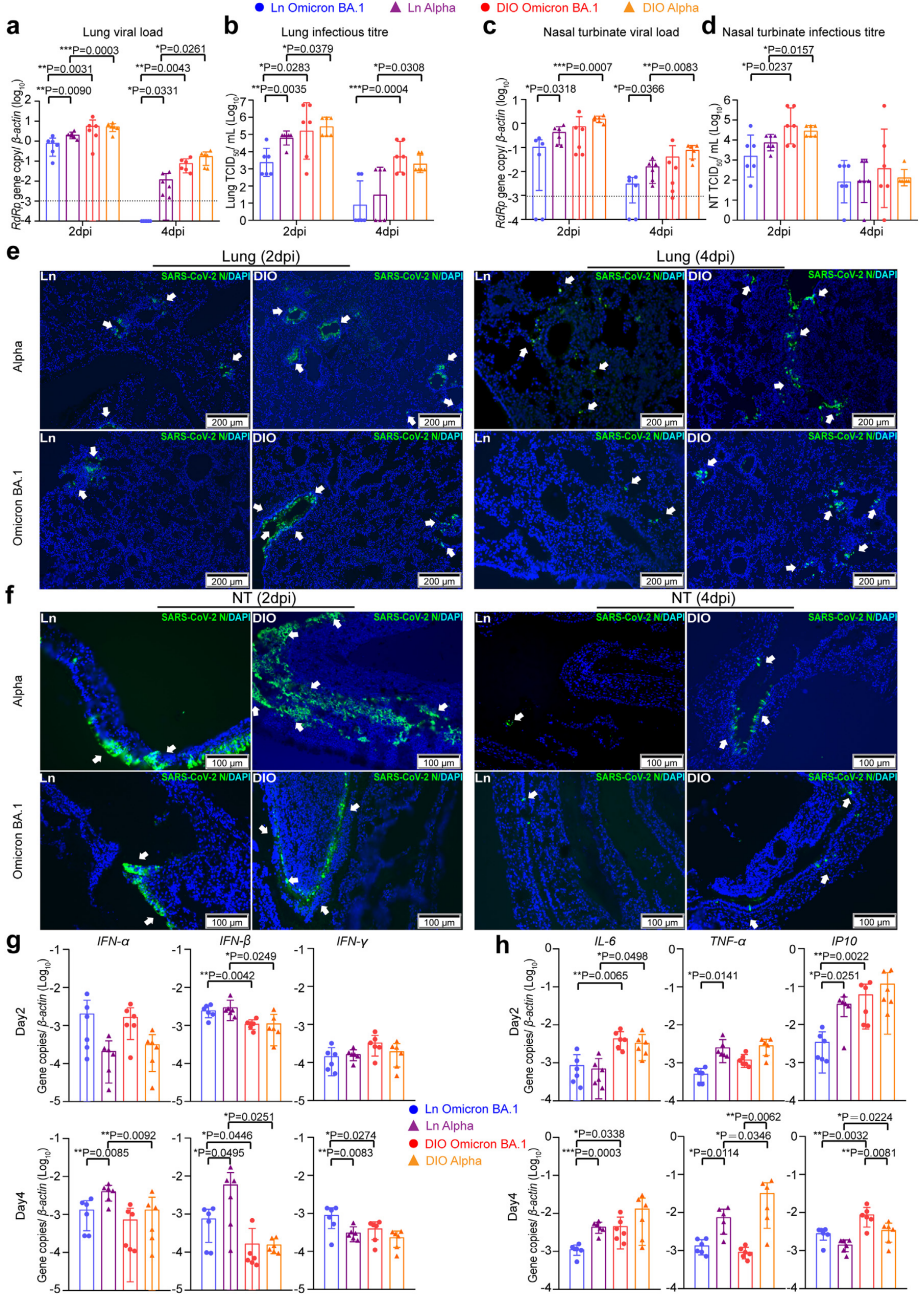
**SARS-CoV-2 replicates more efficiently in DIO mice than in lean mice.** DIO and Ln mice were intranasally inoculated with 10^3^ PFU Alpha or Omicron BA.1. Lung and NT tissues were collected at 2 and 4 dpi for virological analysis and qRT-PCR. **a**–**d** RNA-dependent RNA polymerase (RdRp) gene copies were quantified by RT-qPCR in lung (**a**) and nasal turbinate (NT) tissues (**c**) at 2 or 4 days post infection (dpi) (n = 6). Dashed lines represent detection limit of the assays. Infectious virus titre in lung (**b**) and NT tissues (**d**) were quantified by 50% tissue culture infection dose (TCID_50_) on VeroE6-TMPRSS2 cells (n = 6). **e** and **f** Representative images of immunofluorescence staining of nucleocapsid (N) protein in lung (**e**) and NT tissues (**f**) infected with Alpha or Omicron BA.1 at 2 dpi and 4 dpi. SARS-CoV-2 N was stained in green color and indicated with white arrows. Cell nuclei were stained in blue color with 4′, 6-diamidino-2phenylindole (DAPI). Images in (**e** and **f**) were representative images from 6 mice. **g** and **h** Inflammatory cytokines, chemokines and interferons in lung homogenates of Ln and DIO mice infected with Alpha or Omicron BA.1 at 2 dpi and 4 dpi were quantified by qRT-PCR (n = 6). Data represented means and standard deviations from the indicated number of biological repeats. Statistical significance between groups was determined with one way-ANOVA (**g** and **h**), two-way ANOVA (**a**–**d**). ∗ represented p < 0.05, ∗∗ represented p < 0.01, ∗∗∗ represented p < 0.001, ∗∗∗∗ represented p < 0.0001. Scale bar in (**f**) represented 100 μm and (**e**) represented 200 μm.

We next asked whether Omicron BA.1 and Alpha replicate more effectively in the respiratory tissues of DIO mice in comparison to that of lean mice. We found that both Alpha and Omicron BA.1 replicated to higher levels in the lung tissues of DIO mice than in lean mice at 2 and 4 dpi (Fig. 2a and b). In keeping with previous reports, Omicron BA.1 replicated to lower levels than Alpha in the lung tissues of lean mice. In particular, the infectious titre of Omicron BA.1 was 26.1- and 3.8-folds lower than that of Alpha at 2 and 4 dpi, respectively. In contrast, Omicron BA.1 and Alpha replicated to similar levels in the lung tissues of DIO mice by measuring RdRp gene copies and infectious titres (Fig. 2a and b). In addition, we also detected a similar pattern of viral RdRp gene and infectious titre in the NT tissues, where Omicron BA.1 and Alpha replicated to comparable levels in DIO mice but not in lean mice (Fig. 2c and d). By immunofluorescence staining, we detected more prominent viral nucleocapsid (N) expression in the lung and NT tissues of DIO mice infected with Omicron BA.1 or Alpha when compared to lean mice at both 2 and 4 dpi (Fig. 2e and f and Supplementary Fig. S3). In parallel, we quantified the expression of key interferons (IFNs) and pro-inflammatory cytokines in the lung tissues. Our results demonstrated that Omicron BA.1 and Alpha triggered significantly lower levels of IFN-β in DIO mice when compared to their lean counterparts (Fig. 2g and Supplementary Fig. S4). In contrast, pro-inflammatory cytokines including IL-6, and IP-10 were triggered at higher levels in DIO mice by Omicron BA.1 and Alpha infection when compared to lean mice (Fig. 2h). In addition, we evaluated the baseline expression of ACE2 and TMPRSS2 in uninfected lean and DIO mice and found that ACE2 and TMPRSS2 in lean and DIO mice were expressed at comparable levels. However, immunohistochemistry staining performed on ACE2 antigen expression detected higher intensity of ACE2 in the bronchiolar and alveolar epithelium of uninfected DIO mice when compared to lean mice (Supplementary Fig. S5). Together, these findings suggest that the impaired IFN-β expression and higher ACE2 expression in DIO mice may contribute to the higher virus replication and increased pro-inflammatory response, leading to more severe tissue damage in the DIO mice.

### Adaptive immunity acquired from previous SARS-CoV-2 infection inefficiently protects DIO mice

**Fig. 3:**
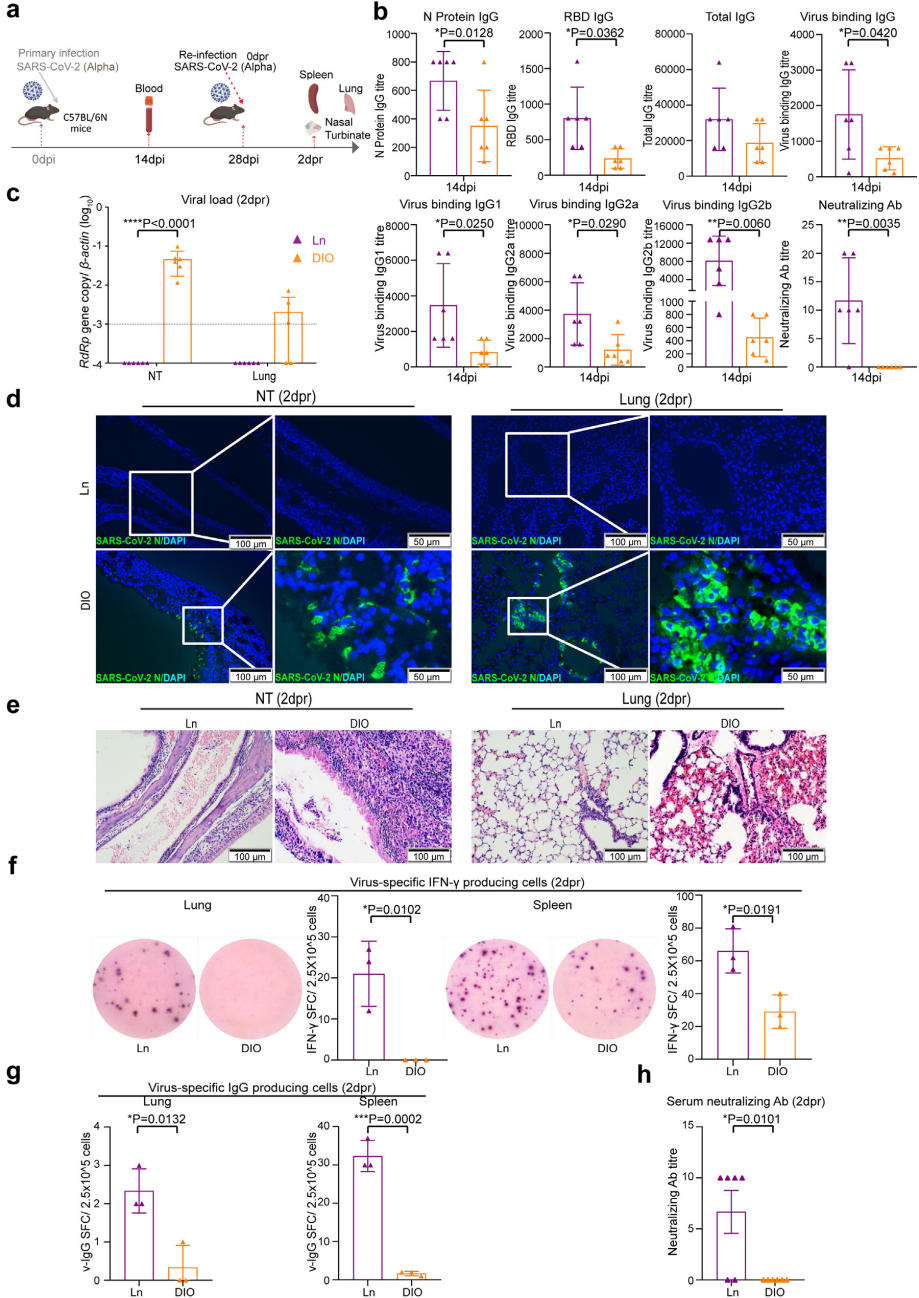
**Adaptive immunity acquired from previous SARS-CoV-2 infection inefficiently protects DIO mice. a** Schematic of primary infection and re-infection of Alpha. After primary infection with 10^3^ PFU of Alpha, mice were re-challenged with the same dose of Alpha at 28 dpi. Blood samples were collected for antibody detection at 14 dpi. Blood samples, spleen, lung and NT tissues at 2 days post re-infection (dpr) were harvested for virological, histological and immunological analysis. **b** Mouse serum of IgG against RBD and N, total IgG, viral binding IgG, and IgG subtype (IgG1, IgG1a, IgG1b) at 14 dpi were determined by ELISA. Neutralizing antibody was detected by microneutralization assay (n = 6). **c** RdRp gene copies of lung and NT tissues at 2 dpr were quantified by probe-specific RT-qPCR (n = 6). Dashed lines represent detection limit of the assays. **d** Representative images of immunofluorescence staining of nucleocapsid protein in NT (left panel) and lung (right panel) tissues at 2 dpr. Squared area were magnified. SARS-CoV-2 N was stained in green color, cell nuclei were stained in blue color with 4′, 6-diamidino-2 phenylindole (DAPI) **e** Representative images of H&E staining for detection of pathological damage of NT (left panel) and lung (right panel) tissues at 2 dpr. Images in (**d** and **e**) are representative images from 6 mice. **f** Virus-specific IFN-γ producing cells in lung and spleen tissues at 2 dpr detected by ELISpot assay (n = 3). **g** Virus-specific IgG-producing cells were detected by ELISpot assay (n = 3). **h** Serum neutralizing antibody at 2 dpr was detected by microneutralization assay (n = 6). Data represented means and standard deviations from the indicated number of biological repeats. Statistical significance between groups was determined with two-tail Student's t-test (**b**, **f** and **g**) and two-way ANOVA (**c**). ∗ represented p < 0.05, ∗∗ represented p < 0.01, ∗∗∗ represented p < 0.001, ∗∗∗∗ represented p < 0.0001. Scale bar in (**d** and **e**) represented 100 μm.

Next, to understand the protective efficiency of adaptive immunity against re-infection in the obese state, convalescent DIO mice and lean mice were re-challenged with 10^3^ PFU of Alpha at day 28 after primary infection and samples were harvested at 2 days post re-infection (dpr) (Fig. 3a). We analyzed the IgG antibody against SARS-CoV-2 antigen in the serum samples taken at day 14 after primary Alpha infection and found that the titre of IgG against SARS-CoV-2 N and spike receptor-binding domain (RBD) were significantly lower in DIO mice than that of lean mice by 1.9- (p = 0.0128) and 3.4-folds (p = 0.0362), respectively (Fig. 3b). In addition, the titre of viral binding total IgG and subtype IgG1, IgG2a, and IgG2b were all significantly lower in DIO mice when compared with lean mice (Fig. 3b). Serum neutralization titre of 1:10–1:20 were detected from 5 out of 6 lean mice but was not detected from any of the 6 DIO mice (Fig. 3b), which suggested an impaired adaptive immune response in DIO mice upon SARS-CoV-2 infection. At 2dpr, viral RdRp gene copy was readily detected from all NT tissues and 3/6 (50%) of lung tissues of re-challenged DIO mice (Fig. 3c), whereas no viral RdRp gene copy was retrieved from the NT and lung tissues of re-challenged lean mice. Consistently, we frequently detected viral N protein in the epithelium of NT and lung tissues in DIO mice but not lean mice (Fig. 3d). Histological examinations revealed epithelial destruction in NT tissues as well as alveolar wall congestion, peribronchiolar infiltration, and localized alveolar hemorrhage in lung tissues of DIO mice but not lean mice (Fig. 3e). Next, we evaluated the recall of immune memory responses upon re-infection in lean and DIO mice, we found that virus-specific IFN-γ and IgG producing cells in both lung and spleen tissues of DIO mice were significantly lower than that of lean mice at 2dpr (Fig. 3f and g). Importantly, we were not able to detect any neutralizing antibody titre against Alpha in the serum of DIO mice at day 2 upon re-infection of Alpha, while 4/6 (66.7%) of re-infected lean mice had a neutralizing titre of 1:10 (Fig. 3h). Thus, our findings suggest that DIO mice are more susceptible to re-infections due to the insufficiently mounted adaptive B cell and T cell immune responses upon SARS-CoV-2 infection.

### COVID-19 mRNA vaccination offers less protection against Alpha infection in DIO mice due to attenuated adaptive immune response

**Fig. 4:**
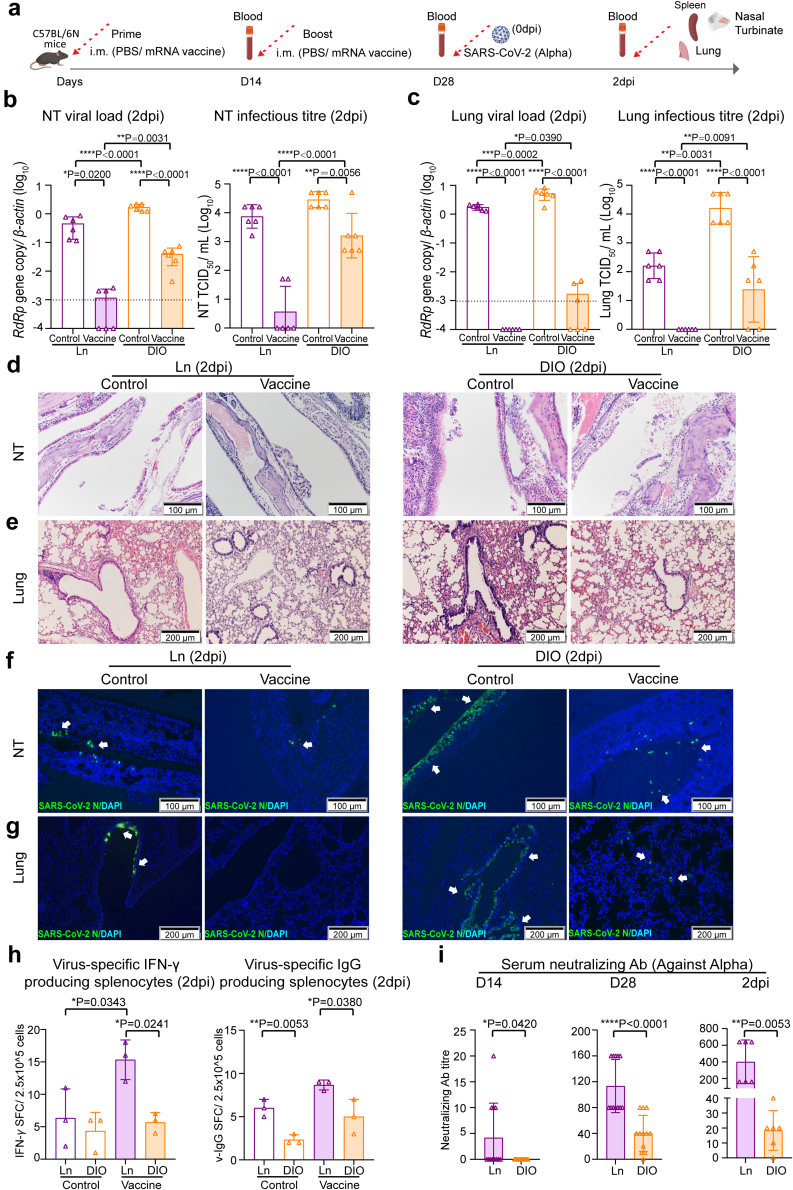
**COVID-19 mRNA vaccination offers less protection against Alpha infection in DIO mice due to attenuated adaptive immune response. a** Schematic of vaccination schedule and virus infection of Alpha. DIO and Ln mice were intramuscularly vaccinated with two doses of COVID-19 mRNA vaccine (5 μg of antigen per mouse) or normal saline as control at a 14-day interval. Blood was collected at 14 and 28 days after primary vaccination. Mice were intranasally inoculated with 10^3^ PFU of Alpha at 14 days post secondary vaccination. Blood samples, lung, NT and spleen tissues were harvested at 2 dpi for immunological, virological and histological analysis. **b** and **c** RdRp gene copies of NT (**b**) and lung (**c**) tissues at 2 dpi were quantified by RT-qPCR and infectious titre of NT (**b**) and lung (**c**) tissues were determined by TCID_50_ assay (n = 6). Dashed lines represent detection limit of the assays. **d** and **e** Representative images of H&E staining of NT (**d**) and lung (**e**) tissues at 2 dpi. **f** and **g** Representative images of immunofluorescence staining of N protein in NT (**f**) and Lung (**g**) tissues at 2 dpi. Images were representative images from 6 mice. SARS-CoV-2 N was stained in green color and indicated with white arrows, cell nuclei were stained in blue color with 4′, 6-diamidino-2 phenylindole (DAPI). **h** Virus-specific IFN-γ (left panel) and virus-specific IgG (right panel) producing cells of spleen tissues were detected by ELISpot assay (n = 3). **i** Serum neutralizing antibody responses against Alpha at 14, 28 days after primary vaccination and 2 dpi were determined by microneutralization assay. (n = 12 for 14, 28 days after primary vaccination, n = 6 for 2 dpi). Data represented means and standard deviations from the indicated number of biological repeats. Statistical significance between groups was determined with two-tail Student's t-test (**i**) or one-way ANOVA (**b**, **c**, and **h**). ∗ represented p < 0.05, ∗∗ represented p < 0.01, ∗∗∗ represented p < 0.001, ∗∗∗∗ represented p < 0.0001. Scale bar in (**d** and **f**) represented 100 μm, in (**e** and **g**) represented 200 μm.

To explore the efficiency of protection for DIO mice after immunization with mRNA vaccines, we intramuscularly injected DIO and lean mice with a two-dose COVID-19 mRNA vaccination regimen followed by Alpha challenge at 14 days post boost as illustrated in Fig. 4a. For NT tissues, we detected viral RdRp gene in 3/6 (50%) and infectious titre in 2/6 (33.3%) of vaccinated lean mice, while we detected viral RdRp gene and infectious titre in all vaccinated DIO mice (Fig. 4b), suggesting that breakthrough infection post-vaccination occurred significantly more readily in DIO mice in comparison to the lean mice. In keeping with previous reports that suggested vaccinations offer better protection of the lower respiratory tract when compared to the upper respiratory tract in human and animal models,^43^^,^^44^ we did not detect viral gene copy or infectious virus from any of the lung tissues of vaccinated lean mice (Fig. 4c). In contrast, we detected viral RdRp gene in 3/6 (50%) and infectious titre in 4/6 (66.7%) of lung tissues of DIO mice. Histologically, we observed extensive epithelium destruction in NT tissues of vaccinated DIO mice after challenge by Alpha virus, while a lesser degree of NT tissues damage was observed in vaccinated lean mice (Fig. 4d). In the lung tissues of vaccinated DIO mice, we detected congestion and infiltration in alveolar space accompanied by foci of alveolar hemorrhage upon Alpha infection, while these histopathological damages were largely absent in lung tissues of vaccinated lean mice (Fig. 4e). In line with these findings, COVID-19 mRNA vaccination reduced SARS-CoV-2 N expression in both lean and DIO mice in the NT tissues (Fig. 4f). In lung tissues, viral N signals were only detected in vaccinated DIO mice but not vaccinated lean mice (Fig. 4g). Next, we found that the virus-specific IFN-γ and IgG producing cells in the spleen tissues of vaccinated DIO mice were significantly lower when compared with that in vaccinated lean mice at day 2 after Alpha infection (Fig. 4h). Importantly, microneutralization assay only detected neutralizing antibody against Alpha in the serum of DIO mice at 14 days after second dose of vaccination and 2 days after virus challenging, but the level was 2.8-folds (p < 0.0001) and 21.8-folds (p = 0.0053) lower than that of the lean mice, respectively (Fig. 4i). Collectively, these results indicate that adaptive antibody responses to COVID-19 mRNA vaccination are impaired in DIO mice.

### COVID-19 mRNA vaccination ameliorates lung damage caused by Omicron BA.1 challenge in DIO mice in the absence of detectable neutralizing antibody

**Fig. 5:**
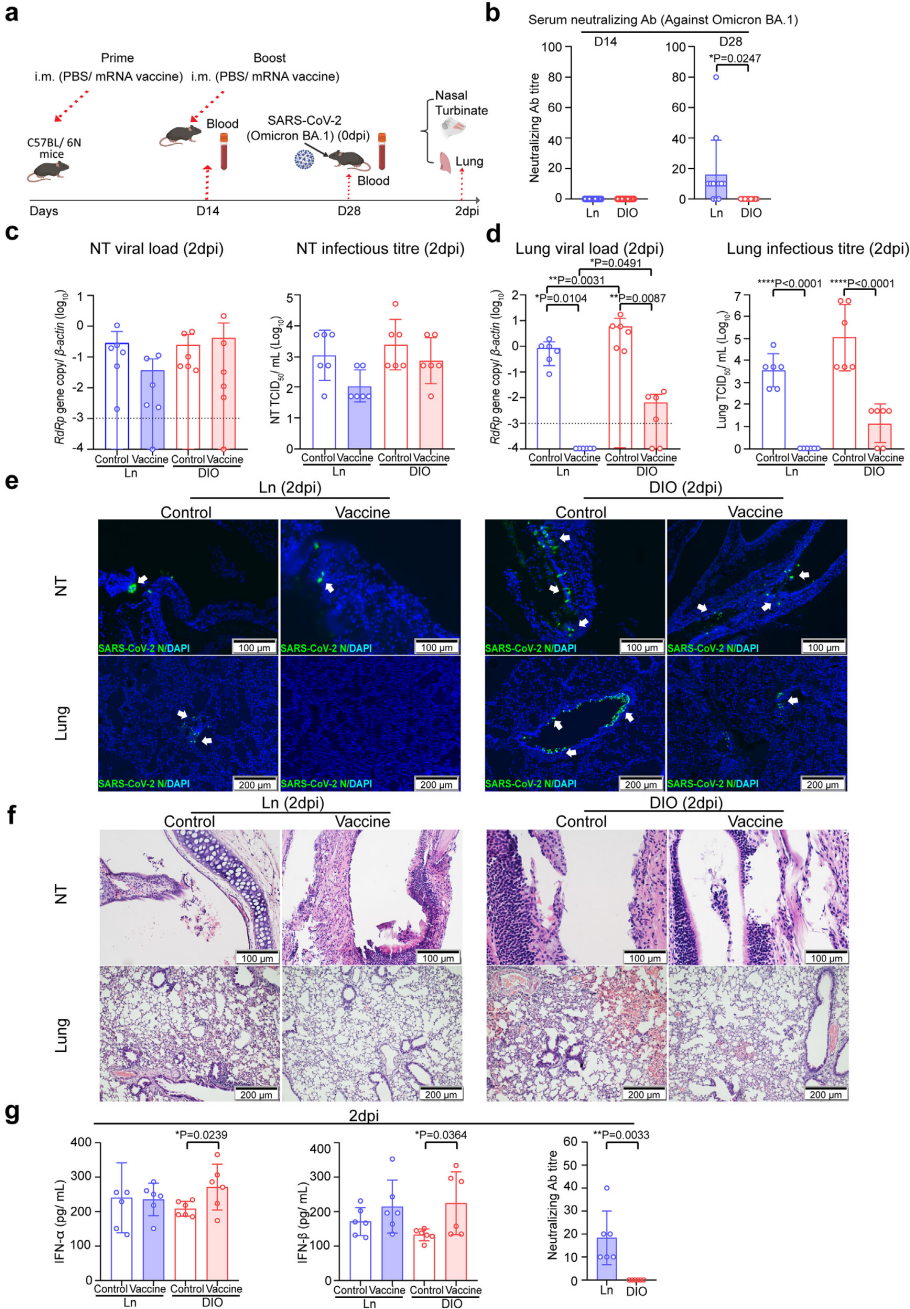
**COVID-19 mRNA vaccine ameliorates lung damage in the absence of neutralizing antibody response in Omicron BA.1-infected DIO mice.a** Schematic of vaccine schedule and virus infection of Omicron BA.1. DIO and Ln mice were intramuscularly vaccinated with two doses of COVID-19 mRNA vaccine (5 μg of antigen per mouse) or normal saline as control at a 14-day interval. Blood samples were collected at day 14 and day 28 after primary vaccination. Mice were intranasally inoculated with 10^3^ PFU of Omicron BA.1 at day 14 post secondary vaccination. Blood samples, lung, NT tissues were harvested at 2 dpi for immunological, virological and histological analysis. **b** Serum neutralizing antibody responses of DIO and Ln mice against Omicron BA.1 at 14, 28 days after primary vaccination were determined by microneutralization assay (n = 12 for 14 and 28 days after primary vaccination). **c** and **d** RdRp gene copies of NT (**c**) and lung (**d**) tissues at 2 dpi were quantified by RT-qPCR and infectious titre of NT (**c**) and lung (**d**) tissues were determined by TCID_50_ assay (n = 6). Dashed lines represent detection limit of the assays. **e** Representative images of immunofluorescence staining of N protein in NT and Lung tissues at 2 dpi. SARS-CoV-2 N was stained in green color and indicated with white arrows, cell nuclei were stained in blue color with 4′, 6-diamidino-2 phenylindole (DAPI). **f** Representative images of H&E staining of NT and lung tissues at 2 dpi. Images in **e** and **f** were representative images from 6 mice. **g** Protein concentrations of IFN-α and IFN-β in lung homogenate at 2 dpi were determined by ELISA. Serum neutralizing antibody responses against Omicron BA.1 of vaccinated DIO and Ln mice at 2 dpi were determined by microneutralization assay (n = 6). Data represented means and standard deviations from the indicated number of biological repeats. Statistical significance between groups was determined with two-tail Student's t-test (**b** and **g**) and one-way ANOVA (**c** and **d**). ∗ represented p < 0.05, ∗∗ represented p < 0.01, ∗∗∗ represented p < 0.001, ∗∗∗∗ represented p < 0.0001. Scale bar in (**e** and **f** of NT) represented 100 μm, in (**e** and **f** of Lung) represented 200 μm.

Next, we asked to what extend COVID-19 mRNA vaccination protects DIO mice against SARS-CoV-2 Omicron BA.1. To this end, we first tested serum neutralizing antibody titre against Omicron BA.1, and detected low titre in lean mice at 14 days post boost, while no neutralizing antibody titre was detected in DIO mice even after the two doses of vaccination COVID-19 mRNA boost or 2 days after Omicron BA.1 infection (Fig. 5a and b), suggesting vaccination in DIO mice induced little cross antibody against Omicron BA.1. Next, DIO and lean mice were challenged with 10^3^ PFU of Omicron BA.1 via the intranasal route at 14 days post boost vaccination (Fig. 5a). In the NT tissues, COVID-19 mRNA vaccination modestly reduced Omicron BA.1 replication and infectious titre in lean mice but had little effect in DIO mice comparing to unvaccinated controls (Fig. 5c). Interestingly, COVID-19 mRNA vaccination significantly reduced Omicron BA.1 replication in the lung tissues of DIO mice comparing to their unvaccinated controls, though not as effective as the complete inhibition of Omicron BA.1 in the lung tissues of vaccinated lean mice (Fig. 5d). Specifically, the Omicron BA.1 RdRp gene copy was reduced by 867-folds (p = 0.0087) and infectious titre by 7979.9-folds (p < 0.0001) in vaccinated DIO mice. Consistent with the above virological findings, COVID-19 mRNA vaccination was less effective in reducing Omicron BA.1 antigen expression in NT tissues of lean and DIO mice but reduced Omicron BA.1 antigen expression in lung tissues of lean and DIO mice (Fig. 5e). At 2 dpi, Omicron BA.1 infection resulted in certain degree of congestion and infiltration in alveolar space accompanied with pulmonary hemorrhage and epithelium damage in unvaccinated DIO mice, which were reduced by COVID-19 mRNA vaccination (Fig. 5f). Importantly, we found that the concentration of IFN-α and IFN-β in the lung tissues of vaccinated DIO mice were significantly augmented comparing to the unvaccinated mice at 2 dpi after Omicron BA.1 challenge, while serum neutralizing antibody remained undetectable from the vaccinated DIO mice (Fig. 5g). The overall disease severity by body weight changes showed that COVID-19 mRNA vaccination reduced the maximum body weight loss in DIO mice to 9% when compared with 13% in unvaccinated DIO mice (Supplementary Fig. S6). Taken together, these results indicate that despite triggering an undetectable level of antibody response, COVID-19 mRNA vaccination may enhance the innate antiviral responses to ameliorates Omicron BA.1-induced lung tissues damage in DIO mice.

### COVID-19 mRNA vaccination upregulates antiviral responses in lungs of DIO mice

**Fig. 6:**
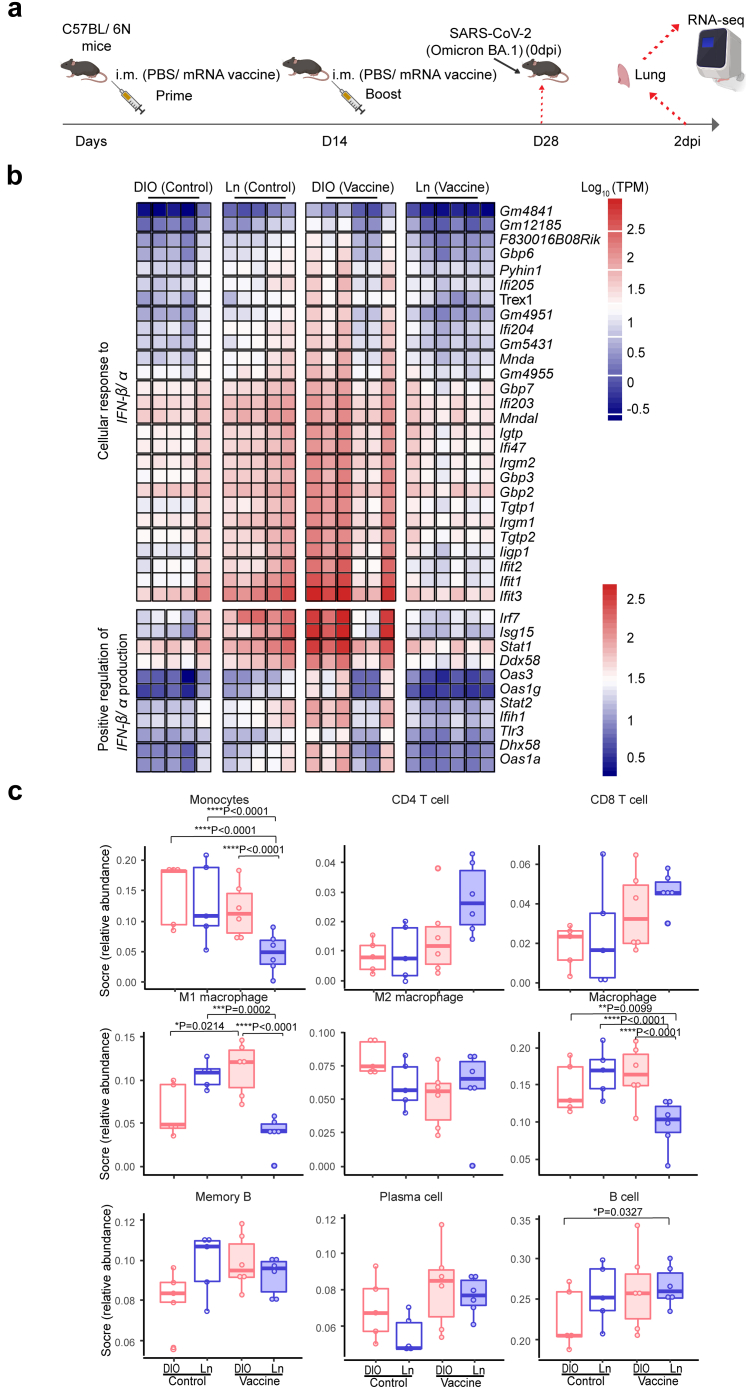
**COVID-19 mRNA vaccination upregulates antiviral responses in lungs of DIO mice. a** Schematic of RNA sequencing experiment design: DIO and Ln mice were intramuscularly vaccinated with two doses of COVID-19 mRNA vaccine (5 μg of antigen per mouse) or normal saline as control at a 14-day interval. After two doses of vaccination, mice were intranasally inoculated with 10^3^ PFU of Omicron BA.1 and lung tissues were harvested from unvaccinated or vaccinated DIO and Ln mice at 2 dpi. **b** Differentially expressed genes (DEGs) in pathways related to the cellular responses to IFN-β/α (GO:0035458 and GO:0035457) and positive regulation of IFN-β/α production (GO:0032728 and GO:0032727) identified by R packages clusterProfile and used for enrichment analysis involving Gene Ontology (GO) and the Kyoto Encyclopedia of Genes and Genomes Pathway (KEGG). **c** Estimation of Immune cell type enrichment by ImmuCellAI Mouse (n = 5 in control unvaccinated group and n = 6 in vaccinated group). Data represented means and standard deviations from the indicated number of biological repeats. Statistical significance between groups was determined with two-way ANOVA. ∗ represented p < 0.05, ∗∗ represented p < 0.01, ∗∗∗ represented p < 0.001, ∗∗∗∗ represented p < 0.0001.

To better understand the immune responses in the lung tissues of DIO mice with COVID-19 mRNA vaccination, we harvested mice lung samples and explored their transcriptome profiles at day 2 upon Omicron BA.1 infection (Fig. 6a and Supplementary Fig. S7). Intriguingly, we observed a notable enrichment of genes involved in pathways related to innate antiviral responses, including “cellular response to IFN-α and IFN-β” and “positive regulation of IFN-α and IFN-β production” in the lung tissues of vaccinated DIO mice (Fig. 6b). Analysis of immune gene sets further revealed that the vaccinated DIO mice had the highest abundance of M1 macrophages among all evaluated groups (Fig. 6c).

**Fig. 7:**
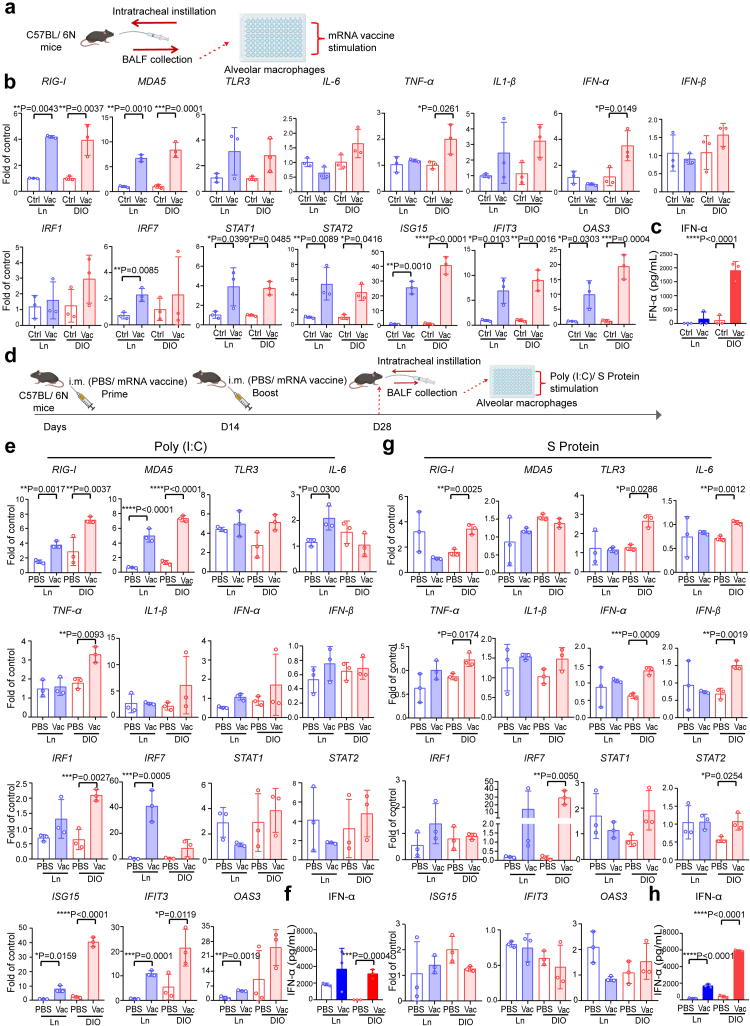
**Alveolar macrophages (AMs) of DIO mice contribute to the upregulated antiviral responses. a** Schematic of AMs from clean Ln and DIO mice stimulation with or without mRNA vaccine. Bronchoalveolar lavage fluid (BALF) from clean Ln and DIO mice were collected by intratracheal instillation with cool PBS, adherent AMs were stimulated with or without mRNA vaccine. **b** Gene expression levels of AMs from clean Ln and DIO mice stimulated with 1 μg/mL mRNA vaccine were quantified by qRT-PCR (n = 3). **c** Protein concentrations of IFN-α in AMs (from clean Ln and DIO mice) supernatants stimulated with 1 μg/mL mRNA vaccine were determined by ELISA (n = 3). **d** Schematic of AMs from unvaccinated or vaccinated Ln and DIO mice stimulation with poly (I:C) or SARS-CoV-2 spike protein. After two doses of COVID-19 mRNA vaccine or PBS, BALF from Ln and DIO mice were collected by intratracheal instillation with cool PBS, adherent AMs were stimulated with poly (I:C) or SARS-CoV-2 spike protein. **e** Gene expression levels of AMs from vaccinated or unvaccinated Ln and DIO mice stimulated with 100 μg/mL Poly (I:C) were quantified by qRT-PCR (n = 3). **f** Protein concentrations of IFN-α in AMs (from vaccinated or unvaccinated Ln and DIO mice) supernatant stimulated with 100 μg/mL Poly (I:C) were determined by ELISA (n = 3). **g** Gene expression levels of AMs from vaccinated or unvaccinated Ln and DIO mice stimulated with 100 ng/mL spike protein were quantified by qRT-PCR (n = 3). **h** Protein concentrations of IFN-α in AMs (from vaccinated or unvaccinated Ln and DIO mice) supernatant stimulated with 100 ng/mL spike protein were determined by ELISA (n = 3). Data represented means and standard deviations from the indicated number of biological repeats. Statistical significance between groups was determined with one-way ANOVA. ∗ represented p < 0.05, ∗∗ represented p < 0.01, ∗∗∗ represented p < 0.001, ∗∗∗∗ represented p < 0.0001.

To evaluate if COVID-19 mRNA vaccine boosted antiviral interferon responses in alveolar macrophage, we isolated alveolar macrophages from lean mice and DIO mice and stimulated with mRNA vaccine (Fig. 7a). We found that mRNA vaccine in vitro stimulation of mouse alveolar macrophages induced significant upregulation of RIG-I, MDA5, STAT1, STAT2, ISG15, IFIT3, and OAS3, while only the alveolar macrophages from DIO mice showed significant enhancement of IFN-α production on mRNA and protein levels (Fig. 7b and c). These findings suggested a differential response profile of alveolar macrophage from DIO mice and lean mice. Next, we isolated mouse alveolar macrophages from vaccinated or unvaccinated lean and DIO mice, followed by stimulating the cells with poly (I:C) or SARS-CoV-2 spike protein (Fig. 7d). In vitro stimulation with poly (I:C) further increased the expression of innate immune response related genes in alveolar macrophages from vaccinated lean and DIO mice. Interestingly, the interferon-stimulated genes including ISG15, IFIT3, and OAS3 were more highly upregulated in vaccine-primed alveolar macrophages from DIO mice when compared to those from lean mice (Fig. 7e). In parallel, recombinant SARS-CoV-2 spike protein stimulation increased the expression of RIG-I, TLR3, IL-6, TNF-α, IFN-α, IFN-β, IRF7, and STAT2 mRNA expression in vaccine-primed alveolar macrophages of DIO mice but not lean mice (Fig. 7g). Importantly, IFN-α production was dramatically increased in alveolar macrophages of vaccinated DIO mice in response to poly (I:C) or S protein stimulation (Fig. 7f and h). Taken together, these results indicate that COVID-19 mRNA vaccination may restore the innate antiviral response in DIO mice lung through modulating the expression of type-I interferon related genes in alveolar macrophages.

## Discussion

Obesity is associated with exacerbated inflammation responses and disruption of immune system that consequently results in a high risk of severity and hospital admission for COVID-19 patients.^45^ It remains unclear how these chronic low-grade inflammation and dysfunction of immune responses modulate the course of SARS-CoV-2 infection. To explore the pathogenesis of SARS-CoV-2 and assess the efficiency of COVID-19 mRNA vaccination in obese individuals, appropriate small animal models are urgently needed to mimic the clinical features of COVID-19 patients. However, ancestral SARS-CoV-2 does not infect wild-type laboratory mouse since RBD of ancestral spike does not efficiently bind to mouse ACE2.^21^^,^^40^^,^^41^ Interestingly, recently emerged SARS-CoV-2 variants, including Alpha (B.1.1.7), Beta (B.1.351), Gamma (P.1), and Omicron BA.1 (B.1.1.529.1), have acquired the N501Y mutation in spike, which enables cross-species transmission of SARS-CoV-2 to wild-type murines.^41^^,^^46^, ^47^, ^48^, ^49^, ^50^ In this study, we use a diet-induced obese wild-type mouse model to investigate SARS-CoV-2 Alpha and Omicron BA.1 pathogenesis, re-infection, and vaccination. Our results demonstrated that Omicron BA.1 infection is less severe than that of Alpha in lean mice according to the bodyweight change, viral load, virus titre, lung pro-inflammatory markers, and histopathology findings. These results are in accordance with recent reports that observed a lower level of infection by Omicron BA.1 compared with previous SARS-CoV-2 variants in animal models.^14^^,^^42^ The increased severity of SARS-CoV-2 infection in DIO mice than in lean mice is in line with results from recent studies performed in diet-induced obese mice and hamsters.^51^, ^52^, ^53^ Interestingly, we revealed that Omicron BA.1 replicated to similar level compared to Alpha in DIO mice, resulting in comparable pathogenicity of Omicron BA.1 and Alpha in DIO mice. In particular, representative pro-inflammatory cytokines including IP-10 and IL-6 were robustly triggered by Omicron BA.1 infection in DIO mice, indicating increased inflammatory damage and morbidity.^54^ Meanwhile, production of antiviral IFN in lung tissues of DIO mice was significantly lower than that of lean mice upon both Alpha and Omicron BA.1 infection. These results suggest that Omicron BA.1 infection in the DIO mice can cause severe disease similar to that of Alpha. It is currently incompletely understood how obesity contributes to severe diseases in COVID-19. The increased leptin level is a hallmark of obesity that regulates metabolism, Jak/STAT and Akt signaling pathways, and modulates T cell functions, which may contribute to the severe outcome.^55^^,^^56^

Antibody-mediated humoral immunity is essential for host defenses,^57^, ^58^, ^59^ while optimal virus clearance in SARS-CoV-2-infected mice also requires CD4^+^ and CD8^+^ T cell responses.^60^ In this study, we detected significantly lower levels of B cell and T cell responses from DIO mice when compared with lean mice, which may contribute to the increased susceptibility of re-infection in DIO mice. The underlying mechanism of the reduced B cell and T cell response in DIO mice remains incompletely explored, but may be associated with impairment of dendritic cell functionality as revealed in previous influenza virus studies.^61^, ^62^, ^63^

Despite the success of COVID-19 mRNA vaccination with high efficacy to protect against SARS-CoV-2 severe diseases, the emergence of immune evasiveness in SARS-CoV-2 variants may jeopardize vaccine efficacy.^43^ In our DIO mouse model with a two-dose immunization regimen, the neutralizing antibody in lean mice were readily detected and robustly boosted by the second dose of vaccination against Alpha and Omicron BA.1. In contrast, the two-dose immunization regimen of COVID-19 mRNA vaccination resulted in undetectable serum neutralizing antibody against both Alpha and Omicron BA.1 in DIO mice, and no neutralizing antibody was detected against Omicron BA.1 even at day 2 upon Omicron BA.1 challenge. These findings indicate that the adaptive B cell responses to mRNA vaccine are severely weakened in DIO mice, which may be due to impairments in B cell development, activation, and functions.^64^, ^65^, ^66^ Interestingly, vaccination offered a certain degree of protection to Omicron BA.1-challenged DIO mice in the lower respiratory but not in the nasal turbinates of infected animals. The insufficient protection in the nasal turbinate tissues may be due to insufficient mucosal immune responses upon intra-muscular vaccination that failed to evoke sufficient protective immunity at the mucosal sites.^67^^,^^68^

Through transcriptomic analysis, we revealed that the COVID-19 mRNA vaccination upregulated antiviral responses in the lungs of DIO mice. Subsequent experiments revealed that IFN-α production in responses to poly (I:C) or SARS-CoV-2 spike protein stimulation was augmented in the alveolar macrophages of vaccinated DIO mice. Together with the reduced viral load and histopathological changes, and increased concentration of IFN-α/β in the lungs of Omicron BA.1-challenged vaccinated DIO mice, our findings suggest that mRNA vaccination boosts the host innate immunity in the obese animals that contributes to the observed protection. Overall, our study reveals important knowledge of SARS-CoV-2 Alpha and Omicron BA.1 infection, re-infection, and vaccination in diet-induced obese mice, which provides insights into the management, treatment, and vaccination strategies for the obese populations.

Our study has a number of limitations. First, we performed re-challenge at 28 days post primary infection. In real-life scenarios, memory longevity and protection of re-infection in patients should be monitored over a longer time frame. Second, female mice were used in this study since females are known to mount stronger innate and adaptive immune responses than males.^69^ The differential immune responses against SARS-CoV-2 between sexes and their roles in disease development of COVID-19 should be further explored. Third, we only used the wild-type mice model for this study, additional evaluation in humans should be performed in future clinical studies to confirm and further explore our findings on whether the COVID-19 mRNA vaccination improves the innate immune responses upon SARS-CoV-2 infection in the vaccinated obese human patients. Nevertheless, our study provides important insights that suggest obese patients may develop more severe clinical diseases upon SARS-CoV-2 infection and are more susceptible to re-infections or vaccine-breakthrough infections. Importantly, our data suggests COVID-19 mRNA vaccines may remain effective in the obese individuals despite low or absence of antibody response, further emphasizing the importance of vaccination in these populations.

## Contributors

AJZ and HC had roles in the study design, data collection, data analysis, data interpretation, writing of the manuscript, supervision, and funding. YXC, WCS, CL, JXW, FFL, ZHY, PDR, YHT, JHL, ZHO, ACYL, PJC, and BHYW had roles in the experiments, data collection, and/or data analysis, JFWC and KYY had roles in experimental protocols and revision of the manuscript. All authors read and approved the final version of the manuscript. YXC, AJZ, and HC have verified the underlying data.

## Materials and correspondence

Correspondence and material requests should be addressed to Dr. Hin Chu or Dr. Anna Jin-Xia Zhang.

## Data sharing statement

The data that support the findings of this study are available from the corresponding authors upon reasonable request. Transcriptomes data is available in CNSA (CNGB Sequence Archive) of CNGBdb under project number CNP0003180 (https://db.cngb.org/cnsa/).

## Declaration of interests

J.F.-W.C has received travel grants from Pfizer Corporation Hong Kong and Astellas Pharma Hong Kong Corporation Limited, and was an invited speaker for Gilead Sciences Hong Kong Limited and Luminex Corporation. K.Y.Y. is the inventor of an intranasal influenza virus-vectored vaccine for SARS-CoV-2.

All other authors declare no competing interests.
