# Supplementary material for: COVID-19 mRNA vaccine protects against SARS-CoV-2 Omicron BA.1 infection in diet-induced obese mice through boosting host innate antiviral responses
Source: eBioMedicine. 2023 Feb 27;89:104485. doi: 10.1016/j.ebiom.2023.104485 (PMC9970285; doi:10.1016/j.ebiom.2023.104485)
Supplement: Supplementary Tables and Figures [file mmc1.docx]

**Supplementary Figures and figure legends**


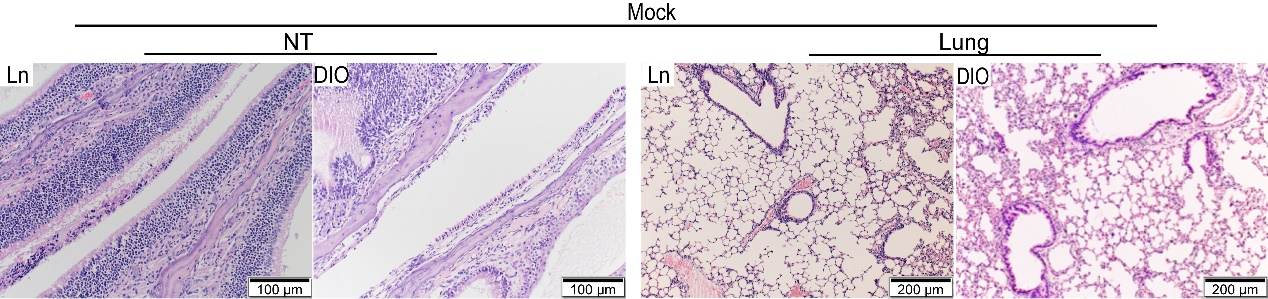


**Supplementary Figure 1. H&E staining of mock-infected mice**. Haematoxylin and Eosin (H&E) staining of formalin-fixed and paraffin-embedding tissue sections (4μm each) from mock-infected mice were observed for histopathology changes. Representative images of H&E staining of NT and lung tissues were shown. Scale bar represents 200 µm for lung and 100 µm for NT.

**
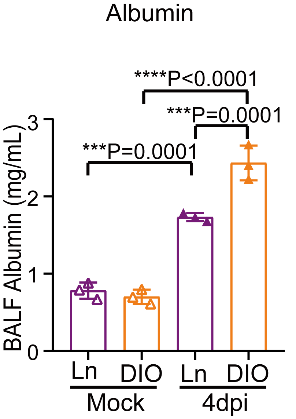
**

**Supplementary Figure 2. Concentration of Albumin in bronchiolar lavage fluid (BALF).** ELISA assay determined the concentration of albumin in BALF of DIO and lean mice with or without infection of Alpha at 4dpi (n = 3). Data represented mean and standard deviations from the indicated number of biological repeats. Statistical significance between groups was determined with one-way ANOVA. *** represented p < 0.001, **** represented p < 0.0001


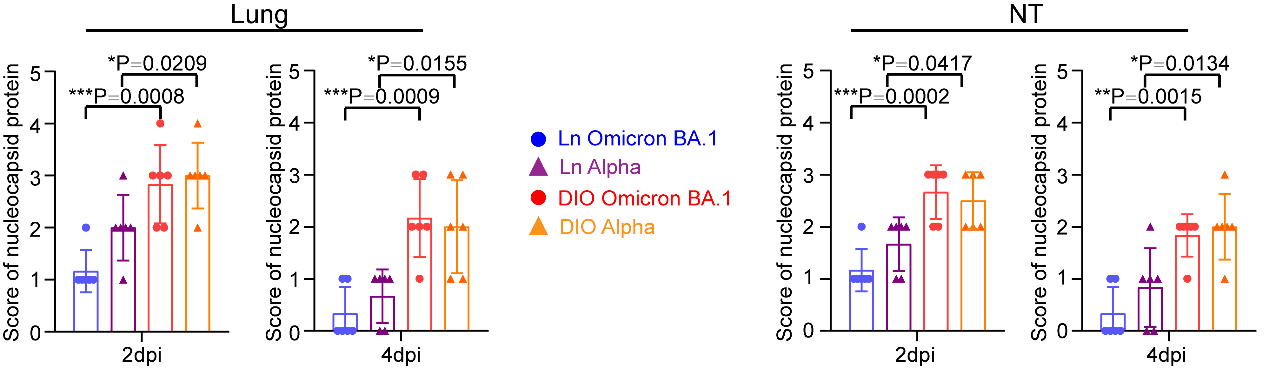


**Supplementary Figure 3. Semi-quantitative of nucleocapsid protein expression in lung and nasal turbinate tissues.** Score of nucleocapsid protein in lung and nasal turbinate sections upon Alpha and Omicron BA.1 infection at 2 dpi and 4dpi. n = 6 in each group. dpi, days post infection; NT, nasal turbinate. Data represented mean and standard deviations from the indicated number of biological repeats. Statistical significance between groups was determined with one way-ANOVA. * represented p < 0.05, ** represented p < 0.01, *** represented p < 0.001.

**
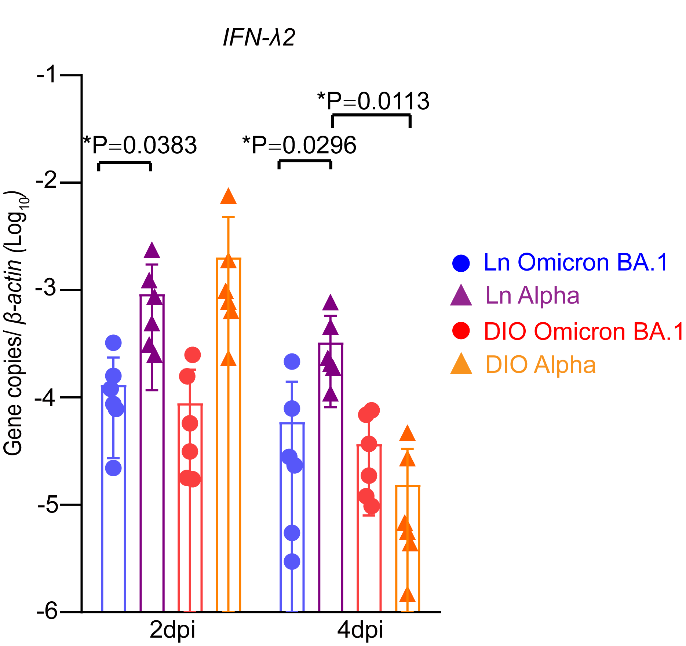
**

**Supplementary Figure 4. IFN-λ2 expression in lung tissues.** IFN-λ2 in lung homogenates of Ln and DIO mice infected with Alpha or Omicron BA.1 at 2dpi and 4dpi was quantified by qRT-PCR. n = 6 in each group. dpi, days post infection. Data represented mean and standard deviations from the indicated number of biological repeats. Statistical significance between groups was determined with one way-ANOVA. * represented p < 0.05.


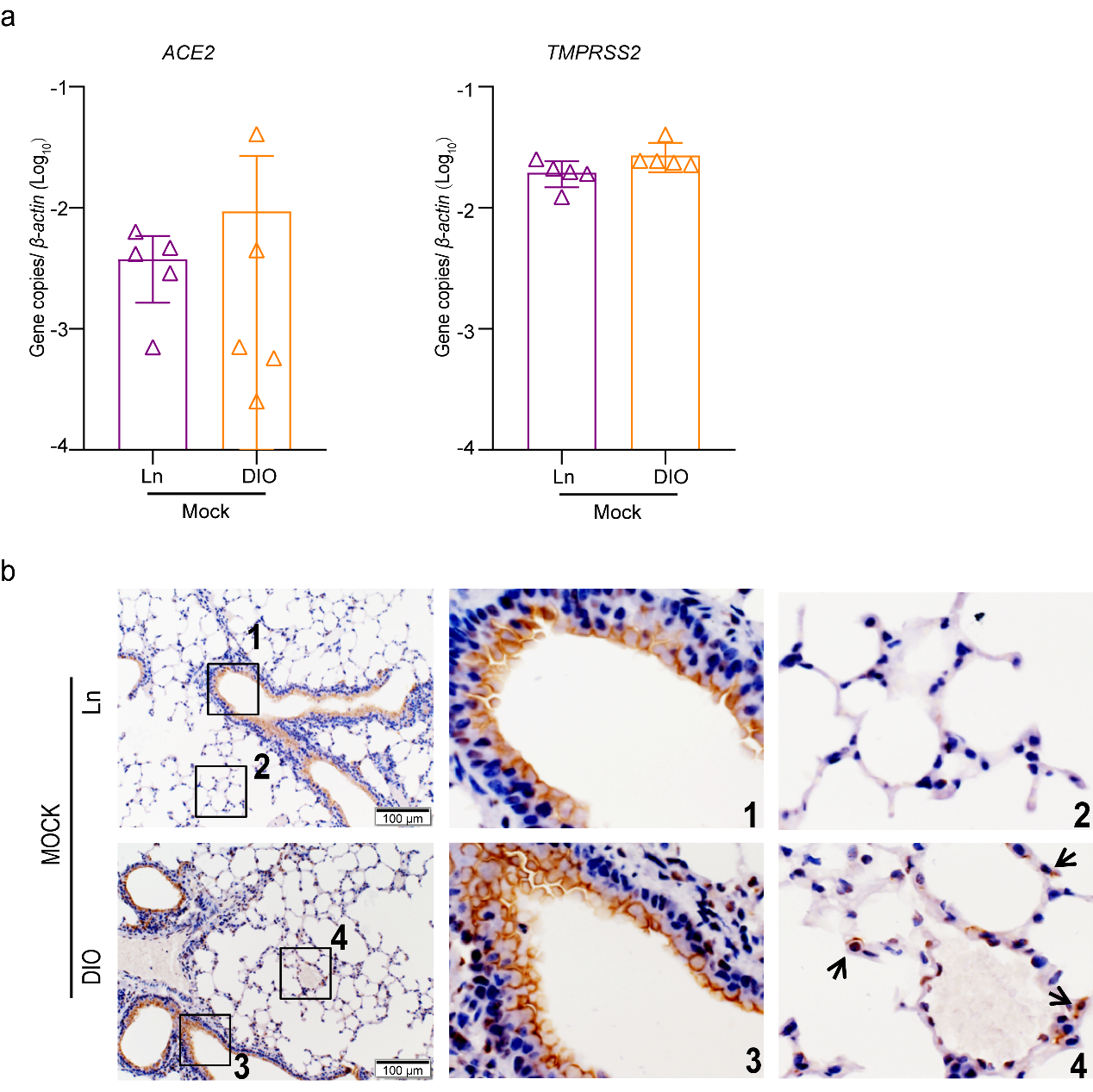


**Supplementary Figure 5. ACE2 expression in uninfected lung tissues. a.** ACE2 and TMPRSS2 in lung homogenates of uninfected Ln and DIO mice was quantified by qRT-PCR. n = 5 in each group. **b.** Representative images of immunohistochemistry staining of ACE2 protein (brown, indicated by black arrows) in the uninfected lung tissues. n = 3 in each group. Data represented mean and standard deviations from the indicated number of biological repeats. Statistical significance between groups was determined with Student’s t-test.

**
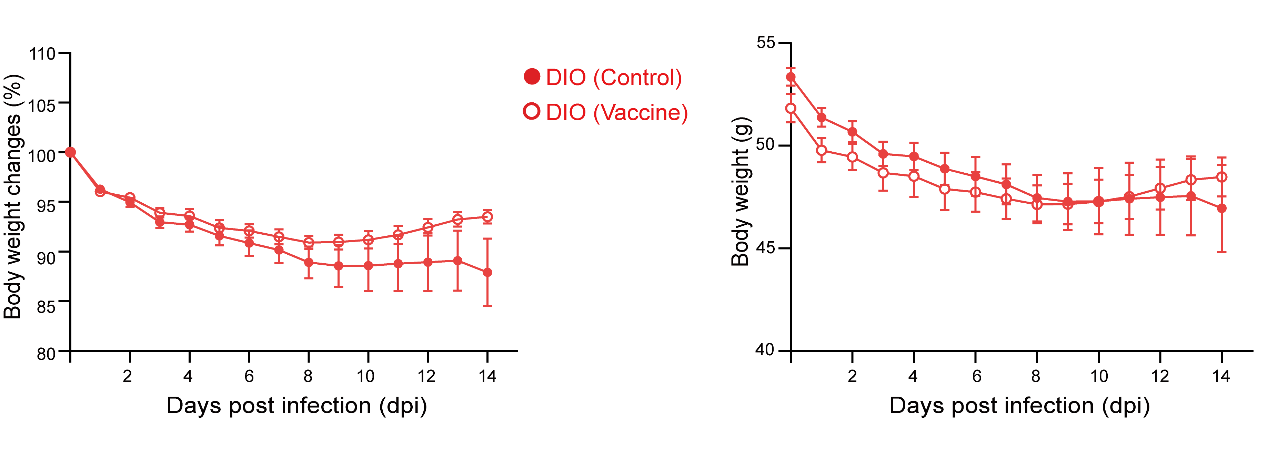
**

**Supplementary Figure 6. Body weight of vaccinated or unvaccinated DIO mice upon Omicron BA.1 infection.** DIO and Ln mice were intramuscularly vaccinated with two doses of COVID-19 mRNA vaccine (5ug of antigen per mouse) or normal saline as control at a 14-day interval and then intranasally inoculated with 10^3^ PFU of Omicron BA.1. Percentage of body weight (left) and total body weight (right) were monitored for 14 days (n = 5). Data represented mean and standard deviations from the indicated number of biological repeats.

**
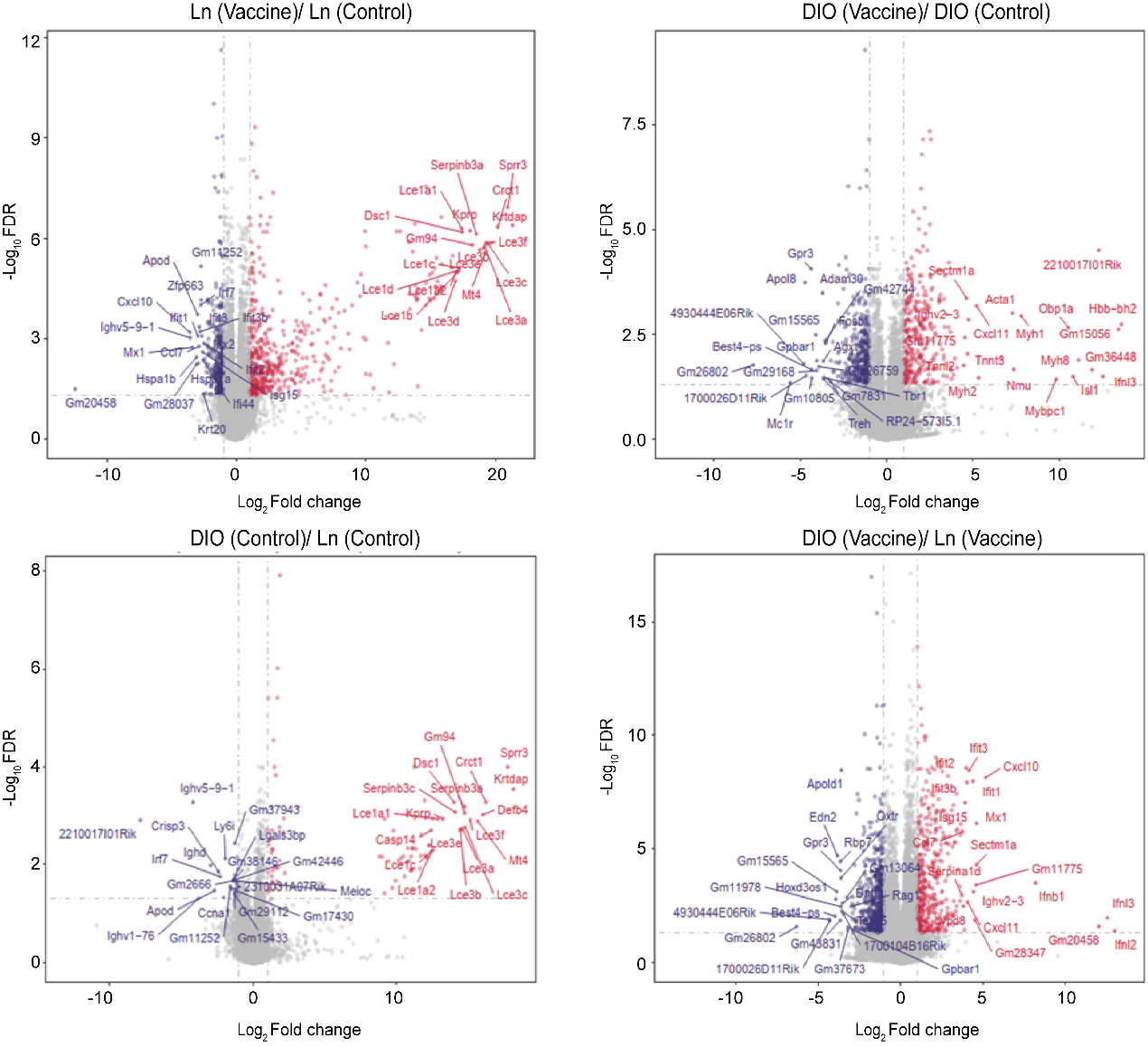
Supplementary Figure 7. Volcano plots of differential expression analysis of mice lung tissues.** DIO and lean mice were challenged with 10^3^ PFU of Omicron BA.1 via the intranasal route at 14 days post boost vaccination. Lung tissues were harvested at 2dpi and used for transcriptomic analyses. The cut-off of dysregulation was set at FDR (adjusted p value <0.05) and |log_2_(FC)| > 1.

| Gene name | Forward primer (5’ to 3’) | Reverse Primer (5’ to 3’) |
| --- | --- | --- |
| *SARS-CoV-2 RdRp* | CGCATACAGTCTTRCAGGCT | GTGTGATGTTGAWATGACATGGTC |
|  | **Probe (5’ to 3’)**: FAM- TTAAGATGTGGTGCTTGCATACGTAGAC-lABkFQ | |
| *β-actin* | ATGGCCAGGTCATCACCATTG | CAGGAAGGAAGGCTGGAAAAG |
|  | **Probe (5’ to 3’)**: Cy5-AGCGGTTCCGTTGCCCTGAG-IABkFQ | |
| *IL-6* | TGGAGTCACAGAAGGAGTGGCTAAG | TCTGACCACAGTGAGGAATGTCCAC |
| *TNF-α* | ATAGCTCCCAGAAAAGCAAGC | CACCCCGAAGTTCAGTAGACA |
| *IL1-β* | GCCTTGGGCCTCAAAGGAAAGAATC | GGAAGACACAGATTCCATGGTGAAG |
| *TLR3* | AATCCTTGCGTTGCGAAGTG | GGTTCAGTTGGGCGTTGTTC |
| *IFN-α* | ARSYTGTSTGATGCARCAGGT | GGWACACAGTGATCCTGTGG |
| *IFN-β* | TGGGAGATGTCCTCAACTGC | CCTGCAACCACCACTCATTC |
| *IFN-γ* | AAGCGTCATTGAATCACACC | CGAATCAGCAGCGACTCCTT |
| *IP-10* | ATGACGGGCCAGTGAGAATG | GAGGCTCTCTGCTGTCCATC |
| *RIG-I* | GAG AGT CAC GGG ACCCAC T | CGG TCT TAG CAT CTCCAA CG |
| *MDA5* | TGATGCACTATTCCAAGAACTAACA | TCTGTGAGACGAGTTAGCCAAG |
| *ISG15* | CATCCTGGTGAGGAACGAAAGG | CTCAGCCAGAACTGGTCTTCGT |
| *IRF1* | TGTCGTCAGCAGCAGTCTCTC | TTCGGCTATCTTCCCTTCCTC |
| *IRF7* | GAGCGAAGAGAGCGAAGAGG | GGCCCACAGTAGATCCAAGC |
| *STAT1* | GCCTCTCATTGTCACCGAAGAAC | TGGCTGACGTTGGAGATCACCA |
| *STAT2* | GAACCAACTCTCCATTGCCTGG | CGTAAGAGGAGAACTGCCAGCT |
| *IFIT3* | GAACATGCTGACCAAGCAGA | CAGTTGTGTCCACCCTTCCT |
| *OAS3* | TTCTCTGCCAGCTTCGGAAAGC | CTCTGAAGGCAGACTTGTGACC |
| *IFN-λ2* | AGCTGCAGGCCTTCAAAAAG | TGGGAGTGAATGTGGCTCAG |
| *ACE2* | AAGGACTTGTCATGGATGCG | CAGCCTTTGAACTTGGGTTGG |
| *TMPRSS2* | AAGCCCCATACTGACCTCCTC | CAGATGTTGAGGCTTGCGTT |

**Supplementary Tables**

Table 1. Sequences of primers and probes

Table 2.

| REAGENT or RESOURCE | SOURCE | IDENTIFIER |
| --- | --- | --- |
| **Antibodies** | | |
| Rabbit polyclonal anti-SARS-CoV N protein | Chan et al., 2020 | N/A |
| Donkey polyclonal anti-Rabbit IgG, FITC | Jackson ImmunoResearch | Cat#711-095-152, RRID:AB_2315776 |
| Rabbit anti-Mouse IgG (H+L) HRP | abcam | Cat#ab6728,  RRID:AB_955440 |
| Goat anti-Mouse IgG1 H-HRP | abcam | Cat#ab98693,  RRID:AB_10674928 |
| Goat anti-Mouse IgG2a HRP | Invitrogen | Cat#M32307,  RRID:AB_2536643 |
| Goat anti-Mouse IgG2b HRP | Invitrogen | Cat#M32507,  RRID:AB_2536649 |
| AP-Goat anti-Mouse IgG (H+L) | Invitrogen | Cat#626522 |
| Rabbit monoclonal anti ACE2 | Thermo Scientific | Cat#MA5-32307 |
| **Critical Commercial Assays** | | |
| QuantiNova Probe PCR Kit | Qiagen | Cat#208256 |
| MiniBEST Universal RNA Extraction Kit | Takara | Cat#9767 |
| PrimeScript RT Reagent Kit | Takara | Cat#RR037A |
| Mouse IFN-γ ELISpot BASIC Kit | Mabtech | Cat#3321-2H |
| Mouse Albumin ELISA kit | abcam | Cat#ab108792 |
| **Chemicals, peptides** | | |
| mRNA Vaccine (BNT162b2) | BioNTech | Cat#1B004A |
| SARS-CoV-2 RBD peptide pool | Genscript Biotech | Custom-made |
| SARS-CoV-2 Nucleocapsid Protein (NP) | ImmunoDiagnostics | Cat# 41A220 |
| Angiotensin-converting enzyme 2 (ACE2) | Invitrogen | MA5-32307 |
| **Experimental Models: Cell Lines** | | |
| Vero E6 cell line | ATCC | Cat#CRL-1586, RRID:CVCL_0574 |
| **Experimental Models: Organisms/Strains** | | |
| C57BL/6N mice | Centre for Comparative Medicine Research, the University of Hong Kong | N/A |
